# Update of the S2k guideline on the management of IgE-mediated food allergies 

**DOI:** 10.5414/ALX02257E

**Published:** 2021-07-08

**Authors:** Margitta Worm, Imke Reese, Barbara Ballmer-Weber, Kirsten Beyer, Stephan C. Bischoff, Barbara Bohle, Knut Brockow, Martin Claßen, Peter J. Fischer, Eckard Hamelmann, Uta Jappe, Jörg Kleine-Tebbe, Ludger Klimek, Berthold Koletzko, Lars Lange, Susanne Lau, Ute Lepp, Vera Mahler, Katja Nemat, Martin Raithel, Joachim Saloga, Christiane Schäfer, Sabine Schnadt, Jens Schreiber, Zsolt Szépfalusi, Regina Treudler, Martin Wagenmann, Thomas Werfel, Torsten Zuberbier

**Affiliations:** 1 Allergology and Immunology, Department of Dermatology, Venereology, and Allergology, Charité – Universitätsmedizin Berlin, Germany,; 2 Nutritional Counseling and Therapy, Focus on Allergology, Munich, Germany,; 3 University Hospital Zurich, Department of Dermatology, Zurich, Switzerland, and Cantonal Hospital St. Gallen, Department of Dermatology and Allergology, St. Gallen, Switzerland,; 4 Clinic of Pediatrics m. S. Pneumology, Immunology and Intensive Care Medicine, Charité – Universitätsmedizin Berlin, Germany,; 5 Institute of Nutritional Medicine and Prevention, University of Hohenheim, Stuttgart, Germany,; 6 Institute of Pathophysiology and Allergy Research, Center for Pathophysiology, Infectiology and Immunology, Medical University of Vienna, Austria,; 7 Department of Dermatology and Allergology, Biederstein, Klinikum rechts der Isar, Technical University of Munich, Germany,; 8 Klinik für Kinder und Jugendmedizin/Päd. Intensivmedizin, Eltern-Kind-Zentrum Prof. Hess Klinikum Bremen-Mitte,; 9 Practice for Pediatric and Adolescent Medicine m. S. Allergology and Pediatric Pneumology, Schwäbisch Gmünd,; 10 University Clinic for Pediatric and Adolescent Medicine, Evangelisches Klinikum Bethel gGmbH, Bielefeld,; 11 Research Group Clinical and Molecular Allergology, Research Center Borstel, Airway Research Center North (ARCN), member of the German Center for Lung Research (DZL), Borstel,; 12 Interdisciplinary Allergy Outpatient Clinic, Medical Clinic III, University Hospital Schleswig-Holstein, Lübeck,; 13 Allergy and Asthma Center Westend, Berlin,; 14 Center for Rhinology and Allergology, Wiesbaden,; 15 Pediatric Clinic and Pediatric Polyclinic, Dr. von Haunersches Kinderspital, Department of Metabolic and Nutritional Medicine, Ludwig-Maximilians-University, Munich,; 16 Pediatric and Adolescent Medicine, St.- Marien-Hospital, Bonn,; 17 Practice for Pulmonary Medicine and Allergology, Buxtehude,; 18 Paul-Ehrlich-Institut, Langen,; 19 Practice for Pediatric Pneumology/Allergology at the Children’s Center Dresden (Kid), Dresen,; 20 Medical Clinic II, Malteser Waldkrankenhaus, Erlangen,; 21 Department of Dermatology, University Medical Center, Johannes Gutenberg-University Mainz,; 22 Nutritional Therapy, Focus on Allergology and Gastroenterology, Schwarzenbek, Germany,; 23 German Allergy and Asthma Association, Mönchengladbach, Germany,; 24 Pneumology, University Hospital of Otto von Guericke University, Magdeburg, Germany,; 25 University Hospital for Pediatrics and Adolescent Medicine, Medical University of Vienna, Austria,; 26 Clinic of Dermatology, Venereology and Allergology, University Medical Center Leipzig, Germany,; 27 Nose and Throat Clinic, University Hospital Düsseldorf, Germany,; 28 Clinic of Dermatology, Allergology and Venerology, Hannover Medical School, Germany, and; 29 Department of Dermatology, Venerology and Allergology, Charité – Universitätsmedizin Berlin

**Keywords:** food allergy, IgE diagnostics, provocation testing, nutritional counseling, therapy

## Abstract

Not available.

Developmental stage: S2k

AWMF guideline register number: (061-031)

Completion: June 2021

Validity: Until December 31, 2024

ICD-10 codes: T78.0, T78.1, L27.2, L23.6, T78.2[Table Abbreviations]

## Preamble 

The 2015 guideline was updated by authors of the chapters after literature searches of PubMed, meta-analyses, clinical trials, and other scientific research. Consensus of the revision was accomplished by an interdisciplinary expert panel. 

It takes into account the methodological guidelines of the Association of the Scientific Medical Societies (AWMF) for the development of guidelines for diagnostics and therapy and corresponds to an S2k guideline according to the three-stage concept of the AWMF [1]. The DELBI criteria were taken into account [2]. 

The strengths of the individual recommendations are provided in this guideline by standardized expressions (Table 1) [3]. 

## 1. Epidemiology and most frequent triggers of food allergy 

*M. Worm and U. Jappe*

How are food allergies differentiated according to their sensitization pathway? How common are food allergies? What are the risk factors of food allergy? What is the prognosis of food allergy? What are the most common food allergies? 

**Classification**

Immunoglobulin E (IgE)-mediated food allergies are divided into primary and secondary food allergies, which can vary in severity.

- Primary food allergies arise primarily (most likely) from a gastrointestinal sensitization to predominantly stable food allergens (glyco-/lipo-proteins).
- Secondary food allergy results from sensitization to aeroallergens (e.g., pollen allergens) with subsequent reactions (so-called cross-allergies) to structurally related, often unstable allergens in (plant) foods.

**Prevalence of food allergies**

The prevalence of food allergies varies from region to region and has increased in some countries in recent years. For example, the prevalence of peanut and tree nut allergy has tripled in the United States in recent decades [4]. Recent data from a European prevalence study, involving Switzerland, but not Germany and Austria, confirm previous data on the frequency of food allergy [5]. Food allergy leads to a reduction in the quality of life of those affected and, in rare cases, can be fatal [6]. 

In order to determine the incidence, prevalence, current developments, potential risks and prognostic factors of food allergy in Europe, studies from 2000 – 2012 regarding this question were reviewed in a meta-analysis [7]. The point prevalence of self-reported food allergy was up to 6 times higher than food allergy verified by provocation testing. The prevalence of primary food allergy was higher in children than in adults. The increase in the incidence of secondary food allergy due to cross-reactivity with inhalant allergens is also due to increased awareness and improved diagnostics. 

Studies on the epidemiology of food allergy in Germany are limited. A study from 2004 found a prevalence of food allergy, confirmed by double-blind, placebo-controlled food challenge of 3.7% in adults [8] and 4.2% in children [9]. A study of adult health in Germany (DGES), conducted in 2008 – 2012, found a lifetime prevalence of food allergy of 6.4% in women and 2.9% in men and for the total cohort of adults of 4.7% (95% confidence interval 4.1 – 5.4) [10]. 

**Factors influencing the frequency of food allergy**

The frequency of food allergy depends on several factors:

- Age and gender,
- family history of atopy,
- place of residence/geographic location,
- dietary habits,
- the presence of other allergic diseases.

Geographically, the prevalence is highest in children compared to adults in North-Western Europe. A lower frequency of self-reported and confirmed food allergy was found in Southern Europe. However, data on the frequency of food allergy should be interpreted with caution, because of the heterogeneity of studies regarding methodological or diagnostic differences within and between (different) geographic region(s) of Europe. 

The frequency of food allergy is difficult to determine for several reasons:

- Presence of augmentation factors (factors that favor the occurrence of food allergy symptoms),
- poor reproducibility of described symptoms,
- relevance of hidden allergens or novel foods,
- consideration of individual sensitization profiles,
- natural development of tolerance

**Prognosis**

Data on the course of food allergy show that early milk protein allergy has a good prognosis in terms of spontaneous tolerance development, whereas peanut and tree nut allergies may persist into adulthood. Further studies are needed to better define the long-term prognosis of food allergy in the future. 

Food allergy can be fatal in very rare cases. This mainly affects children and adolescents with peanut and tree nut allergy, but also milk protein allergy [11, 12]. 

**Main triggers of food allergy according to age**

**Figure 1.**
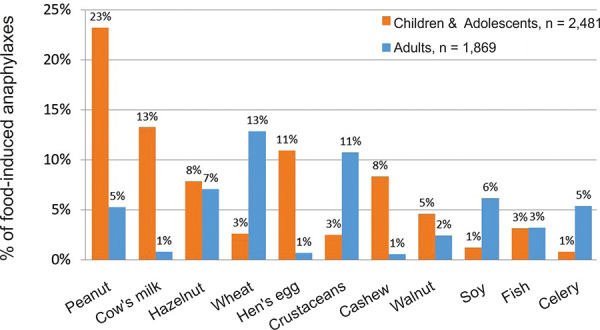
The most frequent triggers of food-induced anaphylaxis. Anaphylaxis Registry; as of March 2019; total food-induced anaphylaxis n = 4,350 (n = 2,481, children and adolescents 0 – 17 years; n = 1,869, adults 18 years and older.

The most common triggers of food allergy in children and adolescents are milk and hen’s egg, soy, wheat, peanut, and tree nuts, and in adults pollen-associated food allergen sources (apple and other pome and stone fruits including hard-shelled fruits, see also Table 7), vegetables (celery, carrot), crustaceans and wheat. The profile of food allergens as triggers of severe allergic reactions is shown in Figure 1. [Table Consensusstatement1]

## 2. Prevention of food allergy 

*K. Beyer and I. Reese*

What measures can be used to influence or reduce the development of food allergy? 

Primary prevention aims at reducing the risk for the occurrence of allergic sensitization and allergic diseases. For this purpose, either causative or predisposing factors are changed or the tolerance of the individual is increased. In the prevention of allergic diseases, a few recommendations apply exclusively to at-risk individuals in whom the father, mother, and/or siblings are already affected by an allergic disease. Most recommendations apply equally to non-risk individuals. 

The German S3 guideline [13] on allergy prevention is currently being updated. In the systematic literature search for this guideline, all allergic diseases and not explicitly food allergy were considered. Since the prevention of food allergy is now also in the focus of preventive approaches, the results of a systematic review of the EAACI were considered for the current revision of this guideline, which forms the basis for the current European recommendations [14]. 

A comparison of the German and European recommendations is shown in Table 2. The German recommendations, which were consented for the prevention of food allergy, also consider the prevention of other allergic diseases, whereas, the European recommendations of the EAACI focus exclusively on the prevention of food allergy in infants and young children. 

At this point, only the consented recommendations for the targeted introduction of potent food allergies are presented and explained. Sufficient evidence exists for the foods hen’s egg and peanut. 

**Hen’s egg**

Regarding hen’s egg, favorable effects were shown by early introduction of cooked hen’s egg [15] or high-heated egg powder [16], whereas administration of pasteurized whole egg was associated with the risk of anaphylactic reactions [17, 18], but showed no advantage for the intervention group [17, 18, 19]. Because baked hen’s egg is thought to have a similar effect to hard-boiled chicken egg or high-heat egg powder, the introduction and regular administration of heated-through****egg (baked, hard-boiled) with complementary feeding is recommended. This includes adequately baked egg-containing baked goods (such as hard cookies, bread and roll specialties, and muffins and cakes). In contrast, it is not recommended to introduce “raw” hen’s egg (including scrambled and soft-boiled eggs) with complementary feeding. 

**Peanut**

The EAACI recommendation for targeted introduction of peanut products for countries with high peanut allergy prevalence was not adopted, as Germany is not currently classified as such. 

Since infants with atopic dermatitis from families with regular peanut consumption are at increased risk of developing peanut allergy, targeted introduction of peanut products in an age-appropriate form (not whole or in pieces because of the risk of aspiration) followed by regular administration may be considered in this constellation. Due to the fact that to date there are only data on the preventive introduction of peanut in infants with mild or no sensitization in the skin prick test to peanut [20], it is recommended that peanut allergy be ruled out in infants with moderate to severe atopic dermatitis before targeted introduction of peanut. 

In addition to the recommendations of the S3 guideline, there is evidence that antacid use may promote sensitization and expression of food allergy [21, 22]. [Table Consensusstatement2]

## 3. Clinical symptoms and differential diagnosis of food allergy 

*L. Lange, B. Koletzko, M. Raithel, and S.C. Bischoff*

### 3.1 Clinical symptoms 

What are the (most common) symptoms of food allergy? 

Depending on

- the ingestion (site of exposure) of the food protein,
- the underlying disease,
- the frequency and type of exposure, and
- the dose

different symptoms of IgE-mediated food allergy can be elicited [23, 24]. Most symptoms are not exclusive to food allergy and may also result from other diseases or non-IgE mediated allergy types. 

Contact of food proteins with the immune system happens most commonly via the oral/gastrointestinal mucosa, but can also occur

- via the skin (e.g., contact urticaria), e.g., as a sensitization pathway for peanut allergy [20]
- the respiratory tract (via the respiratory system, e.g., baker’s asthma, see 7.) or
- via the vascular system (e.g., in the case of contamination of injection solution with food proteins).

The route of exposure plays an important role in the outcome of clinical symptoms. Depending on the organ system involved, various symptoms – often in combination – can occur (modified according to [25]) (Table 3 and 4). In seropositive IgE-mediated allergies with positive IgE detection on skin and/or in blood (often atopy), variable symptom patterns consisting of extraintestinal and intestinal symptoms are found. Most frequently, skin and mucous membrane symptoms occur, for example, as urticaria or angioedema. In severe food allergies, respiratory and/or cardiovascular symptoms may occur. In children, respiratory symptoms are more common (e.g., wheezing or dyspnea) in adults, cardiovascular symptoms are more common. Interestingly, gastrointestinal symptoms are not more common in systemic food allergy. In seronegative IgE-mediated food allergy, only localized IgE in the tissues (entopy) can lead to isolated organ reactions (e.g., oral mucosal swelling, etc.) [26, 27, 28, 29, 30]. Although the IgE-mediated response is an immediate reaction, at the gastrointestinal tract (GIT), depending on the site of digestion, resorption, and/or reaction, symptom may be rapid (upper GIT) or delayed for several hours (middle and lower GIT) [26, 27, 29, 31, 32]. [Table Consensusstatement3]

### 3.2 Manifestations and differential diagnoses 

What other diseases can cause the symptoms of food allergy? What are the clinical manifestations of food allergy? 

Food can cause numerous diseases. These are based on different pathophysiological mechanisms with involvement of different, sometimes several organ systems. 

An overview of the manifestations of food allergies and differential diagnoses is given in Table 5. 

**Non-allergic mechanisms**

Food additives and natural flavorings may also activate mast cells and mimic the clinical picture of IgE-mediated food allergy (postulated mechanisms include activation of G protein-coupled receptors, alterations in eicosanoid metabolism, increased mediator formation/secretion). Natural flavoring agents, sulfur compounds, benzoic acid compounds, histamine-containing foods and glutamate have occasionally been described as triggers of non-allergic food intolerance reactions. In addition, augmentation factors may be required, and oral provocations may be negative if these are not considered. 

The importance of salicylate-containing foods in acetylsalicylic acid (ASA) intolerance is unlikely due to a low occurrence of salicylic acid in foods [33, 34]. Avoidance of salicylate-containing vegetables and fruits is not recommended in terms of an anti-inflammatory diet [34]. [Table Consensusstatement4]

## 4. Diagnosis of food allergy 

*J. Kleine-Tebbe*

How can food allergy be reliably diagnosed? 

**Procedure in case of suspected food allergy**

**Figure 2.**
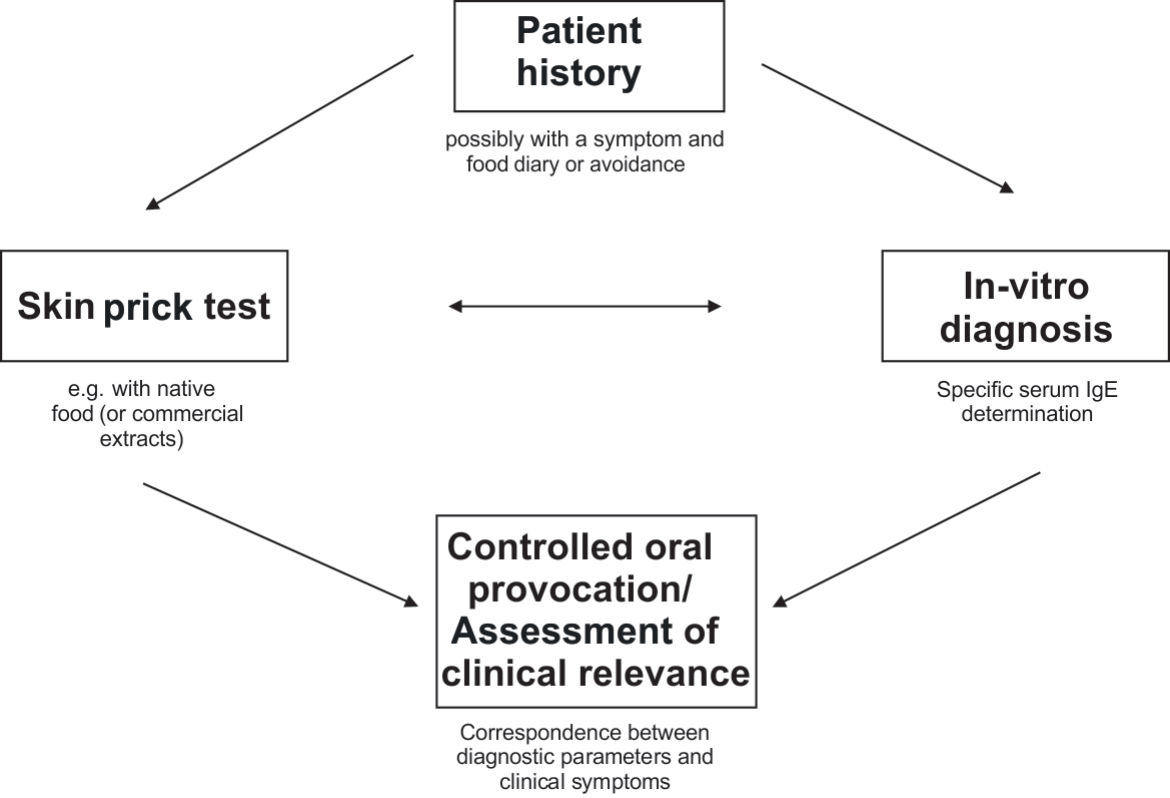
Diagnostic procedure for suspected food allergy: in adults, sensitization is often detected by skin tests (left half), in children preferably by specific IgE determination (right half, see text for additional explanation).

If IgE-mediated food allergy is suspected, the diagnostic procedure is based on several components (Figure 2):

- Patient history (if necessary with dietary and symptom protocol) (4.1.),
- Sensitization test (colloquially “allergy test”)
  - IgE determination (4.2.) and/or
  - Skin prick test (4.3.),
- Determination of clinical relevance (interpretation)
- Plausibility on the basis of the (anamnestic) clinical data,
- If necessary, diagnostic elimination diet and
- Provocation test (4.4.).

The test sequence and the selection of test reagents are based on

- medical history
- the age of the patient and
- the available testing (presented in the subsections).

The diagnostic tests identify increased allergic susceptibility (i.e., sensitization). This is accomplished by:

- direct detection of allergen-specific IgE against food extracts/allergens in serum (4.2.) or through
- positive skin tests (prick test) (4.3.) with food (extracts) as an indirect indication of functional, i.e. capable of cross-linking, allergen-specific IgE on mast cells in the skin.

In principle, the qualitative statements (positive vs. negative) of IgE tests and prick tests are equivalent:

- A negative result serves to exclude sensitization.
- A positive result corresponds to sensitization, which, however, is only clinically relevant in the case of corresponding symptoms.

A single test (IgE test or skin test) may be sufficient to test for sensitization to a food. Multiple tests are often used to detect sensitization (Figure 2). Their results do not always agree qualitatively; in that case, the positive result is more likely to be correct than the (false) negative. In case of concordant results (concordant positive or negative) the diagnostic accuracy is increased, especially since mostly different reagents of a food (native preparations, extracts, single allergens) are used in the skin or IgE test. 

**Interpretation of the tests**

For the interpretation of sensitization tests, the patient history and the clinical symptoms are of central importance: Only if there is a clear agreement between the clinical information of the patient and the test result (prick test/IgE determination), a food allergy can be diagnosed or excluded. If such a match is not or not clearly given (e.g., due to unclear or unproductive patient history), the clinical relevance should be confirmed with oral provocation test (Figure 2) (4.4). 

The term “allergy test” (for skin or IgE tests) is misleading in this context and holds the greatest source of misinterpretation of diagnostic results: A positive result, for example, to food (i.e., sensitization) can only be successfully interpreted if the clinical reaction to a given allergy is known. 

As a rule of thumb, only half of the atopic sensitizations detectable in the population are really associated with symptoms and thus clinically relevant. Thus, sensitization tests show unsatisfactory diagnostic specificity (~ 50%) and limited positive predictive value (“PPV”), which strongly depends on the particular allergen source and the prevalence of food allergy in the cohorts studied. 

In case of gastrointestinal allergy manifestation, specific local diagnostic measures may be considered, such as mucosal or endoscopic provocation and endoscopic lavage. [Table Consensusstatement5]

### 4.1 Medical history and dietary and symptom protocol 

*M. Worm, I. Reese, and L. Klimek*

What is the importance of the patient history in suspected food allergy? 

Which aspects have to be considered in the history of suspected food allergy? 

**Practical procedure for taking the medical history**

The allergy history in cases of suspected food allergy follows basic principles of interviewing. It is helpful to give patients a focused questionnaire before the first appointment, which can be brought to the first interview or filled out during the waiting time. 

The medical history (Table 6) includes:

- the family history regarding atopy,
- the patient’s own medical history, and
- the specific dietary history.

Reported symptoms should be recorded with their local, temporal and situational occurrence. In order to classify the patient’s data, it is important to know whether periods of complete freedom from symptoms occur, but also which foods are usually consumed and tolerated. 

**Supporting measures**

A diet and symptom diary is useful so that patients can monitor their habits and complaints more specifically themselves. Particularly in the case of chronic complaints, records kept by the patient or their parents over 2 – 3 weeks with the aid of a diet and symptom diary are helpful. Such a diary takes into account the intake of food, drinks, but also sweets, chewing gum, etc., special features (e.g., eating in a restaurant) and complaints occurring in a temporal context. Symptom type and intensity should be listed with date, if necessary time, duration of the complaints. The diary should also record medication consumption. The records should afterwards be evaluated by a dietician with experience in allergy or an allergist. By this procedure, the significance of existing (or missing) sensitizations can be critically reflected and the decision for specific provocation tests or other measures facilitated. Furthermore, it should be considered that certain medications (e.g., proton pump inhibitors (PPI) or alkalizing drugs) may favor the development of sensitization [22, 35]. After the diagnostic work up the further therapeutic procedure is planned including a follow-up history. 

**Consideration of augmentation factors**

Augmentation factors should also be considered in the medical history. These can aggravate an allergic reaction and in some cases are even obligatory for triggering symptoms (e.g., in wheat-dependent exercise-induced anaphylaxis). The best known augmentation factors are:

- physical activity and
- the use of non-steroidal anti-inflammatory drugs (NSAID).

However, other factors like alcohol, fever, acute infections, allergic symptoms during pollen season and sleep deprivation [36] have been described as augmentation factors as well [37]. [Table Consensusstatement6]

### 4.2 Triggering allergens and in vitro diagnostics 

*J. Kleine-Tebbe, B. Ballmer-Weber, U. Jappe, J. Saloga, and M. Wagenmann*

How can the severity of a food-related allergic reaction be determined? What are reasonable indications for sIgE determination? What is the significance of diagnostics with single allergens? What is the significance of sensitization to certain single allergens? Which are the most important allergens in food allergy? What must be considered in the interpretation of serological diagnostics? 

**4.2.1 Serological IgE determination for the detection of sensitization**

Allergen-specific IgE in serum against food corresponds to sensitization. A lack of specific IgE (mostly) excludes it, provided that an extract is used for testing in which all important allergens are contained [38]. 

Depending on the test setup, reagents and allergens used, specific IgE results from different manufacturers may differ. 

For IgE testing individual foods (allergen sources, Table 7), a combination of various foodstuffs (search or panel test) and increasingly single allergens (Table 8, 9, 10, further sources of information in Table 11) used [39].****

The diagnostic suitability is evaluated separately according to allergen source, allergen and test method (Table 13). 

*4.2.1.1 Indication for IgE determination*

Depending on

- the age,
- the symptoms and
- and the suspected allergen sources (Table 7)

different indications for in vitro diagnostics are applicable [40]: 

**Suspicion/exclusion of food allergy**

Specific IgE determination is useful in cases of high suspicion or for a specific exclusion of a food allergy. However, this indication requires that all relevant allergens are represented in the test extract used. 

Group tests for specific IgE (e.g., against peanut, fish, hen’s egg white, cow’s milk protein, soy and wheat) allow a rational exclusion or detection of sensitization in the sense of an increased allergic susceptibility. They thus serve as a basis for a further individual allergen source testing. To perform a wide ranged screening without a reasonable suspicion of food allergy is not recommended. 

**Life threatening reactions to food**

In cases of severe anaphylactic reactions, specific IgE determination against the food suspected or to be excluded is preferable and skin testing should be performed according to individual risk-benefit considerations. 

**Suspicion of sensitization to foods not suitable for skin testing**

If the skin test is not suitable as proof of sensitization, a specific IgE determination is recommended (e.g., for skin-irritating foods such as spices). 

**Conditions that do not allow skin testing or its evaluation**

Specific IgE determinations are useful in cases of inadequate skin testing capability. These include urticarial dermographism, skin disease in the test area and medications affecting the skin test. In infants and young children, specific IgE is often determined in serum against allergenic foods instead of skin testing. 

**Common food allergen sources with a low risk potential**

Clinically mild reactions (e.g., oropharyngeal symptoms in pollen-associated food allergy) should be clarified with reasonable effort and in the usual diagnostic sequence (history, skin test, in vitro diagnostics). 

**Example:** If a birch pollen-associated food allergy is suspected, a prick test with a birch pollen extract should be performed and/or a specific IgE test against the main birch pollen allergen Bet v 1. Commercially available fruit or vegetable extracts are often unsuitable for birch pollen-associated food allergy due to unstable allergens. Skin testing with fresh native foods in the prick-to-prick test is more sensitive but less specific. Therefore, an untargeted screening (also serological) of, for example, all fruits and vegetables or the available single allergens in birch pollen-associated cross-sensitization is not recommended [41]. 

*4.2.1.2 Definitions and concepts for allergen selection*

Potential advantages and disadvantages of in vitro diagnostics with extracts or single allergens have to be defined separately for each allergen source or single allergen [42] (information in Table 11). 

The following arguments speak for the use of single allergens:

- Increased test sensitivity [lower limit of quantitation (“LoQ”) [43] by certain single allergens, especially if they are underrepresented or absent in the (food) extract (examples: historically the soy protein Gly m 4 [44], wheat gluten Tri a 19, apple protein Mal d 1, sugar epitope galactose-α-1,3-galactose on proteins and glycolipids in mammalian meat) [45],
- Increased test discriminatory power (analytical specificity or selectivity) for single allergens from allergen sources consisting of complex mixtures of numerous allergens associated with increased clinical risk (examples: Ara h 2 of peanut, Pru p 3 of peach, Cor a 9 and 14 of hazelnut, Act d 1 of kiwi),
- In case of a lack of the analytical specificity of extracts (cross-reactivity), IgE detection against typical cross-reactive allergen molecules facilitates interpretation (examples: Bet v 1 or homologous representative, Phl p 12 or Pru p 4 as profilin, Pru p 3 as lipid transfer protein (LTP), CCD (“cross-reactive carbohydrate determinant”) component MUXF3).

The current limitation of sIgE determination quantity in the reimbursement of IgE diagnostics may lead to an unacceptable limitation of a necessary more extensive screening in unclear cases of food allergy. 

The use of single allergens for IgE determination is mainly justified by their increased test sensitivity (lower LoQ) and (analytical) specificity: If single allergens are thereby able to improve in vitro diagnostics, their use is reasonable and recommendable from an allergological point of view. 

*4.2.1.3 Foods as allergen sources and their allergens*

Foods are complex allergen sources and contain diverse (glyco-/lipo-) proteins, the actual allergens. A relation is thus given by the biological relationship of the foods concerned and by the biochemical similarity of the allergens they contain. The significance of the allergen sources (Table 7) depends on the age of the affected patients and the regional and individual eating habits. 

*4.2.1.4 Important plant protein families and their allergens*

Fruits, vegetables, legumes, tree nuts, oilseeds and cereals can lead to sensitization due to the allergens they contain [46]. 

Meanwhile, the most important protein families and individual allergens of plant foods have been identified (Table 9) and are increasingly used for IgE diagnostics (Table 9, 12). 

**1. Pathogenesis related protein family 10**

Birch pollen allergy, which is common in Central Europe, is predominantly caused by sensitization to the main allergen Bet v 1, a natural plant stress protein (“pathogenesis-related protein family 10”, PR-10). 

Similar PR-10 proteins are present in tree pollen of hazel, alder, beech and oak, as well as in various fruits and vegetables, nuts and legumes (Table 9). They are the basis of birch pollen-associated cross-reactions, for example against apples, cherries, peaches, hazelnuts and many others [41]. Because of the low proportion of PR-10 proteins in the total mass and their lack of resistance to heat and digestion, the symptoms remain restricted to raw foods and mostly to the mouth and throat. In individual cases, life threatening systemic symptoms can also occur, for example, after the ingestion of larger amounts of the consumed food, the presence of augmentation factors such as physical stress, or matrix effects (protection of the PR-10 protein by other food components) [47] (examples: Gly m 4 in soy, Ara h 8 in peanut [48, 49], more rarely Api g 1 in celery, Dau c 1 in carrots). 

**2. Lipid transfer proteins**

Systemic reactions due to fruits, vegetables, nuts, legumes, and cereals can be caused by sensitization to LTP. Predominantly described in the Mediterranean region, primary sensitization possibly arises from ripe peaches [50]. The structural similarity of peach LTP Pru p 3 with the heat- and acid-stable LTP of other plant foods (pome and stone fruits, but also grapes, blueberries, nuts, lettuce) may be responsible for cross-reactions [51]. Meanwhile, more and more cases are observed also in Northern Europe and elsewhere [52]. For the detection of sensitization, the lead allergen Pru p 3 is often sufficient. The clinical relevance of LTP sensitization must be clarified individually with the patient. The patient’s history (clinical reaction) or, in case of doubt, an oral provocation with the suspected LTP-containing foodstuffs serves this purpose. 

**3. Seed storage proteins**

Storage proteins refer to structurally related, yet variable, stable and clinically significant food allergens, for example in nuts, seeds, legumes (*leguminosae*), which include peanut, soybean and lupine, and cereals. 

Based on their structure, 2S-albumins from the prolamin and globulins from the cupin superfamily are distinguished [53]. Globulins contain vicilins (7/8S globulins) and legumines (11S globulins) (Table 9). Due to their stable structure and high proportion of the total protein, storage proteins rarely cause problems in diagnostics with extracts. Due to their stability to heat and digestion, they are often associated with an increased risk of systemic symptoms and are well suited for the identification of sensitization or exclusion:

1. Ara h 2, (if negative, Ara h 1, 3, and 6 if appropriate) in peanut allergy,
2. Cor a 14 (if negative, Cor a 9) in hazelnut allergy,
3. Jug r 1 in walnut allergy,
4. Ber e 1 for Brazil nut allergy,
5. Ana o 3 in cashew and pistachio allergy,

Among the seed storage proteins of wheat, Tri a 19, omega-5 gliadin, is particularly associated with wheat-dependent exercise-induced anaphylaxis (“WDEIA”) [54, 55]. 

IgE detection against storage proteins of nuts, seeds and legumes, do not allow a reliable prediction of the occurrence of clinical symptoms. 

**4. Profilins**

Profilins are phylogenetically highly conserved proteins and are supposed to be clinically less relevant allergens. Sensitizations are often primarily caused e.g., by high grass pollen exposure, can affect all pollens and numerous plant foods (e.g., apples, carrots) and are caused by cross-reactions. In most cases, one representative (e.g., grass pollen profilin Phl p 12, birch pollen profilin Bet v 2 or peach profilin Pru p 4) is sufficient for IgE diagnostics. 

Exotic fruits (e.g., melons, banana, avocado, mango) away from the Bet v 1 food allergen cluster may also have underlying pro lin sensitization as trigger of predominantly oropharyngeal symptoms [41]. Apart from OAS, they may also be responsible for severe allergic reactions in rare cases [56]. 

**Other allergens in plant foods.**

Cross-reactive carbohydrate epitopes: many plant food allergens are glycoproteins with cross-reactive carbohydrate side chains (CCD, including those in pollen, plant foods, arthropods, mollusks, and certain pathogenic helminths). Their IgE binding usually remains without clinical relevance [57]. They do not lead to skin test positivity, but can complicate serological IgE diagnostics with extracts or natural CCD-bearing single allergens by clinically mostly irrelevant results. Bromelain, horseradish peroxidase or the N-glycan MUXF (CCD single allergen component of bromelain without peptide content) are suitable for screening CCD-specific IgE (Table 8). 

**Oleosins: **Oleosins occur in lipid-rich plants as allergens. As lipophilic proteins, they are underrepresented in aqueous extracts of legumes (e.g., peanut), seeds (e.g., sesame), and tree nuts (e.g., hazelnut), and extract-based diagnostics may show false-negative results [58]. In this constellation, testing of native foods by skin testing is indicated. 

**Thaumatins and enzymes:** Thaumatin-related proteins are thermo- and digestion-stable plant food allergens [59], for example in cherries (Pru av 2), apples (Mal d 2), kiwi (Act d 2), banana (Mus a 4), peach (Pru p 2), tomato, bell pepper and walnut. So far, they are only sporadically available for diagnostic purposes (Act d 2, ImmunoCAP ISAC). The frequency of sensitization or clinically relevant reactions is unclear. The same applies to a number of enzymes found in plant foods (e.g., exotic fruits). 

**Examples of component-diagnostic in given allergens**

**Wheat:** Wheat is a relevant food allergen in both childhood and adulthood. Its prevalence has been reported to range from 0.4 to 4% [60, 61]. The sensitizations are more frequently not clinical relevant in children [62]. Baker’s asthma or wheat-dependent exercise-induced anaphylaxis are important clinical pictures of wheat allergy in adults. Since the total extract of wheat gives often false positive results, partly due to strong cross-reactivity to grass pollen, with underrepresentation of other allergens, single allergen component determination is recommended. The most frequently described single allergen is omega-5-gliadin, which, along with other gliadins, may indicate an exercise-dependent wheat allergy. In this case, an allergic reaction is often only triggered after consumption of larger amounts of wheat in combination with physical activity and/or other cofactors [63]. Wheat LTP (Tri a 14) is also an important marker of wheat allergy and presumably not cross-reactive with pollen [64]. 

**Celery:** Sensitization to celery is frequently associated with cross-reactivity to birch pollen and less frequently to mugwort pollen. Several allergens, both in celery stalk and bulb, have been described, e.g., a Bet-v-1 homologue (Api g 1) and an nsLTP (Api g 6) in celeriac stalk****, but also an nsLTP (Api g 2) in celery stalk****[65]. The symptomatology of celery allergy can vary from mild to anaphylactic reactions. Severe clinical reactions to celery have been described in the presence of concomitant mugwort pollen allergy, although the allergen responsible for this is currently not known [64]. 

*4.2.1.5 Common animal food allergens*

Animal proteins from diverse allergen sources can also cause sensitization to foods. They are often stable to heat and digestion and usually responsible for systemic allergic reactions. 

Their structural similarity causes serological cross-reactions within a protein family, however the clinical relevance cannot be concluded from the test result. Due to the complex sensitization patterns and good representation of the proteins in a given extract, a diagnosis with the extracts only is often sufficient. 

**Hen’s egg:** The major allergens in the egg white have been identified (Gal d 1, 2, 3, 4) [66]. 

Sensitization to the major allergen Gal d 1 is associated with persistent hen’s egg allergy due to its heat resistance. If IgE is no longer detectable during the course of a hen’s egg allergy, this may indicate incipient tolerance. Despite clinically relevant egg allergy (even in the case of Gal-d-1 sensitization), a large proportion of affected individuals tolerate egg in baked form [67]. 

**Cow’s milk:** Complex sensitization patterns against predominantly stable cow’s milk proteins and their good representation in cow’s milk extract are reasons for using the total extract for diagnostics [68]. Certain single allergens such as Bos d 8 (casein) are associated with persistent cow’s milk allergy and reactions to processed milk (products) due to their stability. A decreasing or absent IgE may be an indication of incipient tolerance. Cow’s milk in processed form may also be tolerated by a large proportion of cow’s milk allergic patients. 

**Meat:** Allergies to mammalian meat, especially to raw or insufficiently cooked meat products, may result from sensitization to serum albumin. Due to the high cross-reactivity, IgE determination against a representative serum albumin (e.g., Fel d 2 of cat, Bos d 6 of cow) is sufficient. 

Another source of allergic reactions after meat consumption is a carbohydrate epitope (CCD) found in mammals but not in primates: α-Gal. The disaccharide is present in proteins as well as probably in glycolipids and can cause delayed urticarial and severe anaphylactic reactions after red meat [69]; poultry meat, however, is tolerated. If meat allergy is suspected, IgE determinations against albumins, against α-Gal (Ro307, ImmunoCAP, ThermoFisher) and the suspected meat species are useful [70]. 

**Fish:** Reactions to fish are often based on a major allergen from the group of parvalbumins (e.g., Gad c 1 from cod, Cyp c 1 from carp), which show a strong homology. Since additional species-specific fish allergens may sensitize, an extract diagnostic with the suspected fish species is recommended [71]. The high stability of most fish allergens to heat and digestion and the large amounts in the total protein explain their hazardous nature: minute amounts can be sufficient to trigger systemic reactions. In the so-called “fish-chicken syndrome”, a clinically relevant cross-allergy between fish and chicken can occur. Parvalbumin, enolase and aldolase have been described as the underlying proteins [72]. 

**Crustaceans, mollusks, and insects: **Tropomyosin, a muscle protein with high cross-reactivity, is considered an important major allergen of crustaceans and shellfish. In addition to the determination of this major allergen (e.g., Pen a 1, tropomyosin of shrimp), the use of extracts of the suspected animal is recommended due to additional allergens [73]. Shrimp can also be a trigger of exercise-induced anaphylaxis [74]. House dust mite allergic individuals with sensitizations to tropomyosin, the minor allergen Der p/f 10, may be allergic to crustaceans. However, this is not regularly the case. In dust mite and insect allergic patients, the trend towards “edible insects” can lead to severe food reactions [75]. 

*4.2.1.6 Interpretation of serological IgE diagnostics*

Specific IgE against food allergens can only be successfully interpreted if the clinical reaction of the patient is known (Table 13). 

The following errors are possible in the interpretation:

- Sensitizations without corresponding symptoms are misinterpreted as allergy.
- Missing or hardly present allergens in the extract may cause false-negative or too low IgE values.
- Laboratory errors can cause both false-negative and false-positive findings.
- Total IgE should be considered when interpreting quantitative IgE concentrations: very high total IgE (e.g., > 2000 kU/L in patients with atopic eczema) is often associated with numerous sensitizations of questionable clinical relevance.
- If total IgE is low (e.g., < 20 kU/L), even low specific IgE values may be diagnostically significant, and detection or exclusion of sensitization may be difficult.

**Conclusion**

Positve specific IgE corresponds to an IgE-sensitization, which only becomes clinically relevant in combination with a clear matching history and/or a positive provocation test. 

**4.2.2 Cellular methods for IgE- dependent sensitization detection**

IgE-mediated sensitization can be detected indirectly using a basophil activation test (BAT). These tests are currently laborious, costly, and so far not established with allergens of any food allergen for in vitro diagnosis of suspected food allergies (e.g., when total IgE is unusually low, < 20, < 10, < 5 kU/L). 

Recent data suggest that in primary NMA, e.g., to peanut or tree nuts, the results of a BAT (at 10 and 100 ng/mL) are capable to distinguish between clinically relevant and silent sensitizations [76, 77, 78]. Approaches to automate the use of the BAT in a labour- and cost-saving manner are currently under way [283]. [Table Consensusstatement7]

**4.3 Skin testing**

*T. Zuberbier and Z. Szépfalusi*

Which skin test procedure is particularly suitable for the diagnosis of food allergy? 

What should be considered in skin testing for the diagnosis of food allergy? 

Skin tests are a central component of the diagnosis of a food allergy. The skin prick test is the preferred method. Diagnostic sensitivity and specificity may vary depending on the material used (extract, native food). It is usually safe and results are available within 20 minutes. 

**Contraindications**

Contraindications to skin testing include:

- Skin disease in the test area,
- Use of medications that affect skin test results (e.g., antihistamines (H1 receptor antagonists)),
- Presence of symptomatic dermographism, and
- A history of a severe anaphylactic reaction to the suspected food (relative contraindication).

**Restrictions on the use of commercial extracts and criteria for their use**

Many commercial food extracts are not standardized for their allergen content. In children with atopic eczema and food allergy for example, milk, egg, or peanuts, skin testing has high diagnostic sensitivity and negative predictive value (“NPV”), but limited PPV. Skin tests with extracts of plant foods (fruits, vegetables) often (though not always) have insufficient test sensitivity and diagnostic sensitivity. Endogenous enzymatic processes lead to a degradation of less stable allergenic proteins in the extract (e.g., Bet-v-1 homologous food allergens). In addition, important allergenic components are sometimes present in low concentrations. In these situations, prick-to-prick testing with fresh food offers an alternative to commercial extracts (Table 14). 

In practice, skin testing with pollen extracts is useful when pollen-associated food allergy is suspected. Commercial solutions can be used for those foods that have shown high test sensitivity and diagnostic sensitivity in food allergy diagnostics based on studies, such as fish extract. In contrast, for fruits, vegetables, and meat, prick-to-prick testing with native food is more sensitive, thus diagnostically more sensitive, but also more nonspecific. 

**Advantages and disadvantages of testing with native material**

Skin testing with native material can also be helpful to test original dishes. On the basis of a skin test, for example with a cooked mixed original dish, it can be estimated whether and how then the possible individual components are to be examined. In addition, the skin test offers the possibility to test the foods processed in the meal with possible changes in their allergenicity. 

The disadvantage of skin testing with native material is the low diagnostic specificity. For example, false-positive results may occur due to the irritative potential of native food. In rare cases, native food can trigger systemic allergic reactions during skin testing. In addition, this test principle is not standardized or can be standardized. 

**Other skin tests and their significance**

Intracutaneous testing with food has no role in clinical practice, since it represents a considerably higher risk and false-positive reactions can occur. Atopy patch testing with fresh food, for example, based on the suspicion that atopic eczema is aggravated by food allergen sources, rarely provides valuable additional information. 

In the future, the use of fresh food in skin testing will become more important, as the number of commercially available extracts is decreasing since they have to be approved as medicinal products due to European legislation. The associated high costs and simultaneously decreasing sales figures lead to the fact that predominantly only the more frequently demanded allergen sources will be offered by manufacturers [41, 79, 80, 81, 82]. An overview of the food test allergens approved in Germany is available at the homepage of the Paul Ehrlich Institute (https://www.pei.de/DE/arzneimittel/allergene/pricktest/pricktest-node.html). [Table Consensusstatement8]

### 4.4 Diagnostic elimination diet and provocation testing 

*L. Lange, S. Lau, I. Reese, and C. Schäfer*

What is a diagnostic elimination diet and how long should it be performed? What is the significance of food allergen provocation testing and how should it be performed? 

**4.4.1 Elimination diet**

A diagnostic elimination diet is a controlled avoidance of food for a specific period of time. It should only last longer than one to a maximum of 2 weeks in exceptional cases, even for chronic diseases such as atopic dermatitis. For non-IgE-mediated reactions, longer periods (4 – 6 weeks) may be required. There is evidence that a longer-term elimination in IgE-mediated food allergy, in case of late symptoms only, may increase the risk for the onset of immediate reactions on reintroduction [83, 84, 85, 86, 87]. Elimination can also support the loss of tolerance if sensitization is still present [88] and should be avoided in these cases. 

A detailed (complete) documentation by means of a dietary and symptom diary over the period of elimination allows verification with regard to dietary errors. The recurrence of symptoms in the case of dietary errors corroborates the suspected diagnosis, while an absence of symptoms questions intolerance and indicates tolerance. 

Following the diagnostic elimination diet in the absence of symptoms or a significant improvement during the diet an oral food provocation test is recommended upon medical supervision. 

If symptom improvement does not occur under a diagnostic elimination diet, the extent of the diet should be carefully reviewed. Either are the symptoms food-independent or not all eligible triggers have been identified and subsequently eliminated, or augmentation factors are influencing reactivity. 

**Use of therapeutic infant formulas during diagnosis**

Non-breastfed infants with suspected cow’s milk allergy require cow’s milk substitutes for the period of diagnostic elimination in the form of extensively hydrolyzed infant formula or amino acid formula, which should be selected individually (see also 5.3.). If the symptoms do not change despite a carefully controlled elimination diet, an allergy to the avoided foods is highly unlikely. In this case, these foods should be re-introduced into the diet to ensure nutrient coverage and avoid unnecessary dietary restrictions. 

**4.4.2 Food provocations**

Controlled oral provocations are usually necessary to confirm the diagnosis of food allergy or to prove clinical tolerance (Table 15). In addition, it has been shown that regardless of the outcome of an oral food provocation the quality of life of a given patient can improve [89]. The procedure for food provocations is described in detail in national (GPA manual: https://www.gpau.de/fileadmin/user_upload/GPA/dateien_indiziert/Zeitschriften/GPA-SH_Nahrungsmittelallergie_oA.pdf) and international guidelines (EAACI, PRACTALL consensus paper). The guideline “Food allergy due to immunological cross-reactivity with inhalant allergens” addresses the specifics of provocations in pollen-associated food allergies [41]. 

**Decision criteria and influencing factors**

The recommendations include various variables that must be considered in order to perform patient-specific individualized provocations:

- Patient selection,
- safety aspects,
- type and amount of food to be administered,
- time intervals between individual administrations,
- criteria for assessment,
- observation periods and
- formulations.

When cross-reactive foods to inhalant allergies are provoked further aspects should be considered:

- possible accumulation effects during pollen flight,
- altered reaction situation due to augmentation factors (physical exertion, infections, medication and alcohol consumption) as well as
- concomittant diseases (e.g., unstable bronchial asthma, mastocytosis).

**Performance and interpretation of oral provocation tests**

Food provocations can be performed open or blind (single- or double-blind). For pollen-associated food allergy, sequential mucosal and systemic provocation may be used. Open oral provocation testing allows a definite conclusion only if the result is negative. Overall, double-blind placebo-controlled food challenge (DBPCFC) is the gold standard for the diagnosis of food allergy. 

A negative food challenge should be confirmed by repetitive administration of the cumulative amount on the following day at the earliest. DBPCFC are time and personnel consuming. In this respect, negative open provocation may be a useful first step to rule out food allergy. In patients with moderate or severe atopic eczema, DBPCFC are preferable to open provocations. DBPCFC should also be performed when symptoms are subjective, delayed, or atypical, or when patients or parents are anxious. In addition, their use is useful in scientific investigations, for example, to demonstrate the appropriate clinical relevance and potency of the different allergens and to determine threshold dose values of the different food allergen sources. The food allergen administered in the provocation should not be identifiable regarding

- taste,
- odor,
- texture and
- form of presentation (consistency, color and shape) against placebo.

The higher the expectation of a patient for a positive response (overanxious or strongly fixated on a food), the more consideration should be given to changing the standard verum to placebo ratio of 1 : 1 towards a higher number of placebo provocations to better identify and avoid nocebo reactions [90]. 

To avoid severe reactions during a provocation, patients receive the appropriate food in a titrated manner, usually with semi-logarithmic increments at time intervals of 20 – 30 minutes. For many foods, such as cow’s milk, hen’s egg, peanut, and tree nuts, the amounts between 3 mg and 3 g (based on the protein content of the administered food) have been shown to be sufficient in clinical practice [91]. 

Food provocations are usually discontinued as soon as clinical objective reactions occur, or are terminated when the last administered amount and a repetitive administration of the total cumulative dose (e.g., on the following day) has been tolerated without clinical symptoms. If subjective symptoms occur, the next dose should be suspended until improvement occurs or the last dose should be repeated. Immediate reactions occur predominantly within 2 hours after the last food intake. Atopic eczema may continue to worsen for several hours or during the next day after food provocation; therefore, skin examination is required the following day. Urticaria and/or angioedema are the most common immediate type reactions, but gastrointestinal, respiratory, or cardiovascular involvement is also common. There are also allergen-specific differences in the frequency of allergic organ-related symptoms; for example, gastrointestinal reactions such as abdominal pain, nausea, and vomiting are commonly seen with raw hen’s egg, but also with peanut [92]. 

**Safety aspects**

For safety reasons, oral provocations should only take place where allergic reactions, including anaphylaxis, can be adequately treated in an age-appropriate manner. Staff should be trained and experienced in early recognition of symptoms and performance of emergency management. Age- and weight-adapted emergency medications that may be required should be noted and kept on hand in the chart, for example, before provocation starts. For patients with non-IgE-mediated reactions, provocations should be adapted to suit individual circumstances. [Table Consensusstatement9], [Table Rationaleoffoodprovocation]

### 4.5 Mucosal food provocations as an experimental approach 

There is a limited experience (especially for children) – also in terms of the number of patients and controls studied – with mucosal food provocations in adults, which are used in cases of isolated gastrointestinal symptoms and suspected local tissue IgE production on mucous membranes of the gastrointestinal tract (esophagus, stomach, duodenum, caecum, sigmoid/rectum) using an appropriate conventional endoscopic or microendoscopic procedure in specialized centers [93, 94, 95, 96, 97, 98, 99, 100]. This experimental approach aims to differentiate between allergy to food and functional gastrointestinal disease. Similar safety issues should be considered as described for oral provocation. 

### 4.6 Alternative diagnostic tests 

*J. Kleine-Tebbe, L. Klimek, V. Mahler, and K. Nemat*

What alternative diagnostic tests are available? 

What is the importance of alternative diagnostic tests to confirm food allergy? 

A number of alternative diagnostic procedures are used by some physicians and alternative practitioners when food-related symptoms are suspected. These fall into two categories: 

1. Tests with questionable theoretical basis, lack of validity, and no reproducibility. These include bioresonance, electroacupuncture, hair analysis, iridology, kinesiology, and cytotoxic food testing (ALCAT test). These methods have not been successfully validated technically or clinically to justify their use [101, 102, 103, 104, 105]. 

2. Tests with accurate measured data but misleading interpretation: determinations of immunoglobulin G (IgG) or IgG4 antibodies or lymphocyte transformation tests with food do not allow differentiation between healthy and diseased individuals [106], neither in food allergy nor in food intolerance. The lack of diagnostic specificity causes many positive findings in healthy individuals. Food-specific IgG or IgG4 merely indicates that the individual has had repeated contact with the food in question and represents a physiological response of the immune system to a foreign protein. Lymphocyte proliferation after stimulation with food and IgG or IgG4 against food in serum may be elevated in allergic individuals. However, both tests are not suitable for individual diagnosis of food hypersensitivity because of their spread and insufficient specificity [105, 107, 108, 109]. 

The use of IgG/IgG4 determinations with food in suspected cases of food allergy or intolerance is also discouraged by the EAACI [108], the American (American Academy of Allergy, Asthma & Immunology, AAAAI) and the Canadian Allergy Society of Allergy and Clinical Immunology (CSACI) [39, 110, 111]. [Table Consensusstatement10]

## 5. Course and treatment of food allergy 

### 5.1 Natural course 

*L. Lange, U. Lepp, Z. Szépfalusi, and E. Hamelmann*

Can a food allergy transmit to spontaneous tolerance? 

For which food is a development of tolerance likely, for which unusual? 

Most primary IgE-mediated food allergies take the following course: 

Onset in infancy and toddlerhood and spontaneous remission sometimes by school age, sometimes in adolescence [25] depending on the food and concomitant disease [112] or cofactors. 

A later onset of primary food allergy to staple foods, nuts, legumes, or seeds in school age and adulthood is rare. Only in fish allergy, the onset at any age is possible. From adolescence onwards, cofactor-dependent allergies to various foods are increasing. (Table 16). 

The natural course of food allergy is strongly dependent on the food source: Cow’s milk [113, 114], hen’s egg [115, 116, 117], wheat [118], and soy allergies [119] tend to spontaneously resolve over the first years of life. In contrast, peanut [120, 121, 122, 123, 124, 125], tree nut [126], fish, and crustacean [127] allergies persist more frequently throughout life. However, tolerance development is also possible in these cases (Table 17). 

Specific IgE antibodies against food can already appear in infancy and early toddlerhood. The values can increase or decrease over time. A drop of sIgE to a given food allergen is often associated with the development of tolerance [125]. Therefore the development of sensitization can be used to decide whether a repeated provocation test is appropriate. Patients with high specific IgE concentrations to a given food allergen are less likely to develop clinical tolerance. Recent data suggest that low specific IgE antibodies, small skin prick test diameters and low atopic dermatitis severity are more likely to be associated with a remission of food allergy [24, 113]. 

Food allergy in adulthood may represent persistence of a childhood form or a de novo onset. The most frequent triggers are apple, peanut, kiwi, hazelnut, peach, cow’s milk, hen’s egg, wheat, fish, and shrimp [128]. More frequent than primary are secondary food allergies due to cross-reactivities of specific IgE against inhalant allergens. In German-speaking countries the birch pollen-associated food allergy is the most frequent manifestation. These food allergies occurring in adolescence/adulthood may persist throughout life [129]. [Table Consensusstatement11]

### 5.2 Therapy 

Food allergy therapy is based on:

- Short-term treatment of acute reactions and
- Long-term strategies to reduce the risk of further reactions.

The latter include dietary therapy and education programs. These education programs are designed to support individuals to avoid allergen sources and to learn proper behavior in case of an accidental contact (e.g., use/administration of emergency medications). New perspectives for achieving clinical tolerance seem to be offered by sublingual or oral immunotherapy. 

**5.2.1 Acute therapy of food allergy**

*U. Lepp, T. Werfel, M. Raithel, J. Schreiber, S.C. Bischoff, and K. Brockow*

What forms of treatment are available for food allergy? When and how are these used? 

**Key questions**

How effective are pharmacologic and non-pharmacologic interventions in treating acute, nonlife-threatening reactions in food allergy? How effective are pharmacologic and non-pharmacologic interventions in the long-term management of food allergic patients? 

**Therapy of acute reactions**

To successfully manage patients with food allergy, a risk assessment for potentially severe reactions is very important. The risk varies in certain subgroups. In particular, patients with

- previous anaphylactic reactions,
- severe and/or non-controlled bronchial asthma, or
- certain underlying diseases (mastocytosis)

show an increased risk. 

The guideline for the management of anaphylaxis describes how to recognise and treat anaphylactic reactions [130]. Emergency medications must be applied immediately in addition to other emergency medical measures (e.g., fluid and oxygen administration, circulation monitoring, ABCD measures). They are used as initial medication with immediate effect to avert the pathophysiological effects of anaphylaxis. These include epinephrine, bronchodilators, antihistamines, and glucocorticosteroids [130]. Intramuscular administration of epinephrine for anaphylaxis is considered the first-line agent [131]. 

There is no solid evidence that antihistamines are effective for respiratory or cardiovascular symptoms. Moveover, a preventive use of antihistamines may mask the onset of early anaphylactic symptoms (e.g., skin flushing), leading to delayed use of necessary epinephrine [130, 132]. 

Besides epinephrine and antihistamines, glucocorticosteroids are applied in the emergency treatment of food allergy according to the above mentioned guideline [130], although no systematic clinical studies for this indication are available [133, 134, 135]. At moderate doses (1 – 2 mg/kg methylprednisolone), they are effective in the treatment of asthma and may counteract protracted or biphasic reactions [136, 137]. 

**5.2.2 Drug (continuous) therapy of food allergy**

Studies on the prophylactic use of mast cell stabilizers have led to different clinical results [138]. Four randomized trials and two non-randomized comparative studies showed that mast cell stabilizers can reduce the onset of symptoms, while three randomized trials found no efficacy. Thus, no uniform recommendation on the use of mast cell stabilizers is currently possible, but a more differentiated approach is required depending on the patient population studied.

- The mechanism of action of so-called mast cell stabilizers such as cromoglicic acid or ketotifen is still unclear. While a reduced disease activity of intestinal symptoms has been described by possible positive effects on the intestinal barrier, negative reports on the action of cromoglycic acid have been reported regarding cutaneous and extraintestinal symptoms [139, 140, 141, 142, 143, 144, 145].
- There are currently no randomized clinical trials for the use of budesonide in IgE-mediated food allergy. The previous recommendations are based on case and expert reports with an extrapolation of the data derived from patients with eosinophilic disease, which is associated with IgE-mediated allergy in ~ 50% [146].

The aforementioned therapeutic options with mast cell stabilizers and budesonide may be considered individually, if appropriate, for exclusively gastrointestinal symptoms when abstinence measures are not sufficient. They should be critically reviewed primarily by gastroenterologists regarding their efficacy [147]. [Table Consensusstatement12]

### 5.3 Long-term management of food allergy 

**5.3.1 Dietary therapy and allergen labeling**

*I. Reese, K. Brockow, S. Schnadt, and C. Schäfer*

How can dietary restriction measures be successfully implemented in everyday life? 

**Aspects of long-term management**

Long-term management of food allergy includes:

- the avoidance of the eliciting food,
- the replacement with suitable foods
- the regular consumption of tolerated foods (especially if sensitization is present), and
- the management of therapeutic interventions in daily life [148].

Avoidance is the most important therapeutic intervention to prevent the elicitation of symptoms. ****

Valid data on efficacy of avoidance are not available due to ethical reasons, because neither randomized controlled trials including individuals without food allergies are possible nor can food allergic individuals be deprived of their dietary therapy. 

Therapeutic elimination diets are tailored to the individual allergological requirements and nutritional needs of the affected person. The requirements, goals and expectations of a nutritional therapy vary largely depending on the age of the affected individual and the antigenic structure (primary vs. secondary food allergy). 

Ideally, the affected individuals receive the therapeutic counseling from an dietitian specialized in food allergies. It is important to take the individual thresholds into account instead of issuing blanket bans. For example, it has been shown that a large proportion of children with cow’s milk and/or hen’s egg allergy tolerate these foods in baked form [149, 150]. Such partial tolerance allows to expand the diet, and the risk of accidental reactions is lower, a beneficial effect on the development of tolerance is promoted if the food in question is regularly consumed in a baked form [151, 152, 153, 154, 155, 156, 157]. A regular administration of minimal, significantly subthreshold amounts of other allergenic foods is probably also favorable for the promotion of tolerance, but currently not supported by published data [54]. 

Individual tolerance to a food allergen may vary among affected individuals and may change individually. This applies to primary but also secondary food allergies which may manifest only seasonally in some affected individuals. Augmentation factors for a food allergic reaction are listed in the section “Medical history” (see 4.1), these factors should be taken into account in nutritional therapy. 

**Cow’s milk (formula) substitution**

Cow’s milk allergy, which occurs in the first year of life, requires a therapeutical diet (extensive hydrolysate, amino acid formulas) to ensure age-appropriate growth and thriving. However, an adequate supply of all nutrients can only be guaranteed as long as the infant is mainly bottle-fed [158]. 

The choice of formula requires an individual approach: Extensive hydrolysates are usually used as the first choice. For patients with severe symptoms (especially also gastrointestinal), amino acid formulas may be beneficial [158, 159, 160, 161]. 

Soy formulas are not recommended for infants younger than 12 months of age. In addition, their use during the first year of life is viewed critically in Germany due to the content of phytoestrogens, phytate, and aluminum [162]. This is particularly relevant at high intakes per kg body weight, i.e., in the first 6 months of life. With predominant nutrient supply via this milk substitute food and still low consumption of other foods, the risk-benefit ratio of soy formula would be unfavorable. 

These limitations are explicitly relevant only regarding soy formula. In contrast, soy products such as soy drink, soy yogurt, tofu, etc. can be used as cow’s milk substitutes. Particularly if these are enriched with calcium, they represent a nutritionally adequate alternative to milk and dairy products. In contrast, other vegetable drinks are not a nutritional substitute due to their low protein and fat content, even when calcium is enriched. 

Partially hydrolyzed infant formulas are generally not suitable for the treatment of cow’s milk allergy, nor are sheep’s or goat’s milk [163, 164]. If a partially hydrolyzed infant formula is tolerated [150], it can be maintained. 

**Food allergen avoidance by the nursing mother**

If breastfed infants suffer from allergic symptoms that are clearly attributed to maternal consumption of certain food allergen sources, the breastfeeding mother should eliminate the suspected or triggering food(s) from her diet after dietary counseling. If milk and dairy products are eliminated long term, the mother should be counseled regarding her nutrient coverage. If adequate coverage cannot be achieved by dietary intake, especially for calcium, supplements should be given throughout the day [165]. 

**Monitoring and reevaluation of clinical relevance**

Extensive and long-term food avoidance should be carefully monitored, as they may result in an inadequate nutrient intake and and impairment of quality of life. 

Consequently, nutritional counseling should cover with a nutriient calculation and, if necessary, an optimization in order to ensure that a given diet is appropriate for everyday life, meets its requirements and is age-appropriate. 

A periodic review of clinical relevance is necessary to ensure that avoidance measures are maintained only as long and to the extent that they are appropriate or necessary. In cow’s milk or hen’s egg allergy, a re-evaluation of clinical relevance by oral food challenge is recommended at (6 –) 12 months intervals in young children and at 12 – 18 months intervals in older children. 

For allergens with a less favorable prognosis, such as nuts and peanuts, the re-evaluation should be determined on an individual level and should be considered especially if no acute allergic reactions have occurred. In pollen-associated food allergies, a repeated follow-up history is supportive to properly assess the clinically relevant cross-reactions over time. 

**Patient education and allergen labeling**

The most important pillar of nutritional therapy is the education of the patient to implement long-term elimination in daily life. 

Patients, their families, relatives and caregivers should:

- Know and be able to identify risk situations,
- learn to read ingredient lists and
- learn to avoid the relevant trigger in an appropriate way both at home and away from home (e.g., in restaurants).

They should be informed about the European Food Information Regulation (FIR [166]):

- The FIR requires the declaration of 14 major triggers of allergies and non-allergic intolerances when they, as well as products thereof, have been added to a food as an ingredient (i.e., intentionally, according to the recipe):
  - Cereals containing gluten (wheat, rye, barley, oats)
  - Crustaceans
  - Egg
  - Fish
  - Peanuts
  - Soy
  - Milk
  - Treenuts (almonds, hazelnuts, walnuts, cashewnuts, pistachios, pecannuts, brazilnuts, macadamia-/queenlandnuts)
  - Celery
  - Mustard
  - Sesame seeds
  - Lupin
  - Mollusks
  - Sulfites (≥ 10 mg/kg)
- Mandatory labeling includes packaged as well as non-prepackaged foods.
- Not regulated by law is the labeling of the unintentional presence of an allergen in packaged or bulk goods. The so-called “trace” labeling is voluntary and, due to the lack of limit values, does not provide any information about the level (allergen quantity) of contamination or its actual probability, nor does its absence per se indicate a safe food. It must therefore always be interpreted individually with regard to its relevance for the patient.

Patients, their families, close relatives and caregivers should be informed

- which substitute products make sense from a nutritional point of view and
- what options are available in terms of kitchen technology to enable the patient to continue to enjoy the familiar dishes and preferences despite avoidance.

**Therapeutic use of pro- and prebiotics**

The use of pro- and prebiotics for the therapy of food allergy is not recommended at present due to limited data. [Table Consensusstatement13]

**Gaps and important areas of research regarding long-term management.**

- Long-term effect of an elimination diet on nutrition and quality of life,
- effect on tolerance development due to altered allergens (baked milk/baked egg),
- effect of strict allergen abstinence in patients who have been shown to develop an allergic reaction only after eating larger amounts of the allergenic food (loss of tolerance),
- long-term disadvantages of rice and soy formulas in terms of nutrient coverage,
- strain-specific (related to specific microorganisms) effects on food allergy management by administration of probiotics or HMOs,
- identification of allergen-specific thresholds. Aim: to protect food allergic individuals from severe reactions and to optimize food labeling with respect to ingredient and “trace” (unintended entry) labeling.

**5.3.2 Immunotherapy for food allergy**

*B. Bohle, K. Beyer, L. Lange, R. Treudler, and M. Worm*

Is immunotherapy efficacious in patients with food allergy? 

**Use of specific immunotherapy (SIT) in food allergy**

Repeated attempts have been made to treat primary food allergy with the aid of

- subcutaneous (SCIT),
- sublingual (SLIT) and
- oral (OIT) specific immunotherapy with food or food extracts. Epicutaneous immunotherapy (EPIT) is a new treatment for food allergy.

Primary sensitizing pollen extracts have been used sublingually and subcutaneously, but also oral and sublingual application of food directly has been studied for the treatment of pollen-associated food allergy. 

The assessment of the efficacy in immunotherapy studies for food allergy is challenging as a standardization of the oral challenge testing, but also the double-blind and placebo-controlled design is required. 

**Use of SCIT in food allergy**

Current data on the efficacy of SCIT for the treatment of food allergy are limited, after this form of treatment for primary food allergy was discontinued decades ago because of severe systemic side effects associated with therapy. 

For SCIT with food allergen extracts, two older studies showed evidence for superiority of treatment with verum over placebo in primary food allergy [167, 168]. However, considerable side effects occurred in this case. Attempts have also been made to develop a hypoallergenic recombinant parvalbumin for SCIT of fish allergy [169]. 

In affected individuals with secondary food allergy, the application of subcutaneously applied pollen allergens has been shown to be effective against pollen-associated food allergy in at least some of the patients in several studies [170, 171, 172, 173, 174]. Here, the effect of SCIT on birch-associated apple, hazelnut, or soy allergy was investigated. Other studies found no efficacy of birch SCIT on birch pollen-associated hazelnut or apple allergy [175]. 

Currently, a peanut allergen extract is in clinical development. Allergoids contain the protein in a partially denatured form, so better tolerability has been shown [176]. 

**Use of SLIT in food allergy**

There are a limited number of studies on the use of SLIT for the treatment of food allergy with single food extracts or recombinant allergens. Daily administration of glycerol-containing liquid allergen extracts under the tongue has been shown to promote desensitization of a food allergen and IgG4 response. For peanut allergy, some studies are available, especially in children/adolescents, showing clinical efficacy with good tolerability [177, 178, 179, 180]. The highest tolerated doses were observed in young children (< 12 years) after a longer duration of therapy (1 year) and are lower than those achievable with OIT. Side effects with this form of therapy are mostly limited to the oral cavity, and systemic reactions are very rare. There are fewer data for other foods. With a hazelnut and Pru p 3-standardized peach extract in 12 and 37 treated patients, respectively, an increase in orally provoked food allergen levels was observed (7.6-fold and 39.8-fold, respectively) [181, 182]. A 16-week SLIT with the main apple allergen Mal d 1 induced an enhanced effect on birch pollen-associated apple allergy than Bet v 1 or placebo [183]. Concomitant immunologic effects on antibody and T-cell response were shown as well [184, 185]. Other studies using a liquid birch pollen extract for SLIT failed to show any or only minor improvements in subjects with apple allergy [186, 187], whereas the SLIT with a standardized allergen tablet containing birch pollen extract was shown to be more efficaceous regarding birch pollen-associated apple allergy in comparison to placebo [188]. 

**Use of EPIT in food allergy**

Epicutaneous immunotherapy (EPIT) has been studied in a phase III trial in patients with peanut allergy, and only in a phase II trial for cow’s milk to date. Here, an application system vaporized with food allergen (e.g., “peanut patch”) is applied to the skin. The dosage is increased by increasing the application time. The patch remains on the skin for 24 hours per day. The duration of therapy in the peanut study was up to 3 years. A moderate increase of the threshold in the peanut provocation compared to placebo was observed over the 3-year treatment. However, in some patients the threshold decreased again. The efficacy of this treatment was most pronounced in children aged 4 – 11 years. The side effects were mild with predominantly skin irritation and only very few treatment-related systemic allergic symptoms [189, 190, 191, 192, 193, 194]. 

**Use of OIT in food allergy**

OIT with a wide variety of food allergens leads to improved tolerance of the food allergen in childhood and adulthood. This has been shown in various randomized and non-randomized controlled trials – mainly with cow’s milk, hen’s egg and peanut, but also wheat [195, 196, 197, 198, 199, 200, 201, 202, 203, 204, 205, 206, 207, 208, 209, 210, 211, 212] – and partly in systematic reviews (“pooled” analyses) based on them [213, 214, 215, 216, 217]. However, side effects occurred in many patients under OIT with the allergen, most of which were not severe. 

Especially for peanut allergy, recent studies suggest that OIT is effective in increasing the threshold of reactions to peanut, reducing the acidic reaction, and increasing the quality of life of those affected [218, 219, 220, 221, 222, 223]. Based on a phase III trial, the first oral immunotherapy was approved in Europe for the treatment of peanut allergy in children aged 4 – 17 years. Data from the USA and Canada also show a good efficacy/side-effect profile for younger children, and a phase III trial for the treatment of peanut allergy in 1 – 3 year old children is currently being conducted internationally. 

An older randomized trial did not show better efficacy with OIT with cow’s milk or hen’s egg compared with elimination diet in terms of tolerance development, however, this trial was conducted in young children in whom natural tolerance development often occurred [224]. Another study showed for cow’s milk allergy that OIT was more effective than SLIT in a direct comparison, but accompanied by more side effects [199]. [Table Consensusstatement14]

**Use of biologics for the treatment of food allergy**

The biologic omalizumab (an anti-IgE antibody) was first used to treat peanut allergy in 2003 [225]. Another trial of anti-IgE use in food allergy occurred years later [226]. Anti-IgE can be applied as monotherapy or as adjuvant therapy to oral immunotherapy for the treatment of food allergy. Numerous case series and prospective studies exist in this regard [226, 227, 228]. Potential benefits include a reduction in the duration of therapy, improved efficacy and tolerability of the allergen dose of OIT, acceleration of the increase phase and improved thresholds, and a reduction in the rate of side effects. Recently, a meta-analysis on the use of omalizumab in food allergy was presented. The data show superiority over placebo treatment both as monotherapy, as adjuvant therapy or for multiple food allergen sources [228]. The 2nd generation anti-IgE antibody ligelizumab has stronger IgE receptor affinity than omalizumab. Studies will show its potential regarding efficacy for the treatment of food allergy in the future. In 2018, the FDA in the U.S. granted omalizumab breakthrough treatment designation and recommended accelerated trials for the approval for severe food allergy. 

Another antibody of great interest for the treatment of food allergy is dupilumab. This antibody targets the IL-4 receptor α chain and has been approved in Germany for the treatment of atopic dermatitis, TH2-dependent bronchial asthma and polyposis nasi. Clinical trials are currently underway to determine its efficacy in food allergy, thereby this antibody may also represent a new treatment option in the future [229]. Etokimab is another antibody that has been reviewed in a randomized placebo-controlled trial as monotherapy for the treatment of peanut allergy [230]. It is an anti-IL-33 antibody that has been used in patients with peanut allergy. A recently published study demonstrated that the etokimab-treated group achieved higher thresholds to peanut protein after 15 days of treatment. 

**5.3.3 Day to day management for at risk of anaphylaxis patients**

*C. Schäfer, S. Schnadt, K. Brockow, and P.J. Fischer*

How can a food-allergic patient successfully manage food allergy in everyday life? 

**Education and risk assessment**

Education and training are the essential components of everyday management for patients with food allergies. Risk assessment is necessary for patients at increased risk for severe allergic reactions. 

Patients, family members and caregivers receive, in addition to emergency medications:

- an individualized nutrition and management plan (see section 5.3.1.),
- an anaphylaxis passport and
- an anaphylaxis emergency plan (see anaphylaxis guideline [130]).

**Emergency plan**

The anaphylaxis action plan should consider all variables that may affect the recognition and treatment of allergic reactions to food:

- Age of the patient,
- Type and extent of food allergy,
- Concomitant diseases,
- Place of residence, and access to medical help.

Procedure management, especially what to do for which symptoms, should be clear to uninformed third parties. 

**Training and anaphylaxis training**

Training should include the following aspects:

- patient-specific avoidance strategies at home and in the social environment,
- recognition and interpretation of warning signs and symptoms,
- when and how to treat allergic reactions, including use of the epinephrine auto-injector.

**Who should be educated on food allergies and anaphylaxis?**

All patients in a trainable state of development caregivers, and, if possible, other family members should be included in the training. In addition, training of other occupationally involved individuals is also appropriate. These include:

- Family physicians and pediatricians,
- other medical specialists,
- medical students,
- medical assistants,
- pharmacists,
- nutritionists,
- psychologists,
- kitchen staff,
- teachers and educators, among others, caregivers of children,
- first aiders in companies,
- paramedics.

In addition to the above-mentioned target groups, there are also other professional groups, such as flight attendants, for whom training may be useful. 

A multidisciplinary approach and the provision of written or online information material on food allergies seem to improve the knowledge and correct use of epinephrine auto-injectors and contribute to the reduction of allergic reactions [231]. In addition to face-to-face training courses, “e-learning” has gained importance as a new training route [232]. 

**Patient organizations**

It is helpful to refer patients to appropriate patient organizations such as the German Allergy and Asthma Association (DAAB – www.daab.de) for questions regarding everyday management. For severe allergic reactions (anaphylaxis), the standardized AGATE training program (Arbeitsgemeinschaft Anaphylaxie – Training und Edukation – www.anaphylaxieschulung.de) is available in Germany. [Table Consensusstatement15]

## 6. Recommendations for vaccination in patients with hen’s egg allergy 

*S. Lau*

Although there is sufficient evidence for the tolerability of the STIKO-recommended vaccinations of the first 2 years of life for children with hen´s egg allergy, MMR varicella vaccinations are repeatedly postponed unnecessarily or not applied due to their production on chicken fibroblasts or insisted on administration in a clinic. 

According to the recommendation of the Robert Koch Institute (as of 2/2020) [233], MMR varicella or MMR vaccination can be administered to hen´s egg allergic patients in a pediatrician’s office. It states on the RKI homepage: “Those who are allergic to chicken egg protein can usually still receive the MMR vaccine, but should seek medical advice before doing so”. 

MMR vaccines are grown on so-called chicken fibroblasts, but the vaccine itself contains little or no detectable traces of chicken protein. 

International studies have shown [234, 235, 236] that children with proven chicken egg protein allergy can be vaccinated with MMR vaccine without problems. Only children who have very severe chicken egg protein allergy with severe symptoms should be vaccinated under special protective measures and observed afterwards (for example, in hospital). 

The indication that only children after severe anaphylaxis (not further specified, but probably grade III and IV) due to chicken egg protein should be vaccinated in hospital or in a specialized practice/day clinic with subsequent observation (2 hours) appreciates the usually great concern of parents and possibly allergologically not so experienced general practitioners. Even in influenza vaccines produced using hen’s eggs, the egg protein content is usually below the dose that normally causes reactions. Therefore, the RKI and the American Center of Disease Control [237] have also published the recommendation to vaccinate patients with rather mild allergic symptoms of a chicken egg allergy normally in practices and to vaccinate only after more severe anaphylaxis under appropriate clinical or specialized outpatient conditions optimized with regard to emergency management, since severe allergic reactions to influenza vaccination are rare or do not occur more frequently than in persons without a hen’s egg protein allergy [238, 239]. However, a chicken protein-free influenza vaccine, i.e., produced in cell culture, is now available that is suitable for allergic individuals. 

The situation is different for the yellow fever vaccine, which contains hen’s egg protein concentrations that can lead to symptoms in ~ 5% of hen’s egg allergy sufferers. Here, the indication should be reserved and only given in case of absolute urgency. The yellow fever vaccine is only administered in specialized vaccination centers and by licensed vaccinators. If an indication exists despite hen’s egg allergy, the vaccination should be given under day-case, clinical, or outpatient conditions with the possibility of emergency intervention, with appropriate follow-up. 

SARS-CoV2 vaccination can also be given to any food allergic patients [240]. [Table Consensusstatement16]

## 7. Food as an occupational allergen 

*V. Mahler*

How common is occupational food allergy and what are the triggers? How is occupational food allergy diagnosed, and what are the consequences for occupational activity? 

**Epidemiology and triggers**

IgE-mediated sensitization to food allergens can also be acquired and clinically manifest via the skin or respiratory tract, which occurs primarily occupationally but also non-occupationally [41]:

- inhalative (work-related) exposure may cause allergic rhinopathy and/or allergic asthma, cutaneous exposure may induce contact urticaria (CU) and/or protein contact dermatitis (PCD) at the site of protein contact (predominantly on the hands) (Table 18) [241, 242] and
- rarely after ingestion [243].

Inhalation symptoms to food allergen sources can lead to the development of an occupational disease (OD) of the lung BK 4301^1^, IgE-mediated cutaneous skin symptoms can result in an OD of the skin BK 5101^2^. 

^1^Obstructive respiratory disease 4301, obstructive respiratory disease (including rhinopathy) caused by allergenic substances, which forced the omission of all activities that were or may be causative for the development, exacerbation or recurrence of the disease.*

^2^Severe or recurrent relapsing skin disease which has compelled omission of all activities which were or may be causative in the development, aggravation or recurrence of the disease.*

*The Seventh Act amending the Fourth Book of the Social Code and other laws, passed by the German Bundestag on 12.06.2020 244. https://www.bmas.de/SharedDocs/Downloads/DE/Gesetze/siebtes-gesetz-zur-aenderung-des-vierten-buches-sozialgesetzbuch-und-anderer-gesetze.pdf?__blob=publicationFile&v=1 (online access: June 28, 2021), which came into force on 01.01.2021, provides in §12 a change in the Annex 1 of the Occupational Diseases Ordinance in the sense that in the occupational disease numbers (BK No.) 1315, 2101, 2104, 2108 to 2110, 4301, 4302 and 5101 the words „... which have forced the omission of all activities that were or may be causal for the development, aggravation or recurrence of the disease“ are deleted. Practical implementation recommendations are currently being developed by representatives of the DGUV and specialist groups.

In the general population, CU and PCD to food allergen sources are very rare; in food processing occupations, the proportion of PCD and CU is significantly higher (1.5 – 20%), depending on the occupation and cohort sample [241, 245, 246, 247]. The prevalence of work-related asthmatic diseases in exposed workers ranges from 1 to 20% and is particularly high among bakers [247, 248, 249, 250]. Flour dust allergy to wheat and rye flour represents the most common cause of occupational allergic obstructive airway disease in Germany [249, 250].

Food allergens from a wide variety of allergen sources have been described as triggers [241, 250, 251, 252, 253]. Asthmatic bakers with sensitization after inhalation exposure to wheat flour show different allergen profiles than individuals with orally acquired wheat-induced food allergy [249, 250]. The extent to which certain food allergens cause specific allergic symptoms depending on the exposure (oral, inhalation, cutaneous) (Table 19) is not yet clear for most allergen sources [249, 254].

**Prevention**

Protection of workers from allergen exposure and sensitization by minimizing occupational health hazards is necessary for the prevention of IgE-mediated skin and respiratory diseases [250, 255]. Occupational dermatology and occupational medicine guidelines and recommendations are available [242, 246, 248, 255, 256, 257, 258, 259]. In order to optimize preventive measures, the responsible statutory social accident insurance should be informed at an early stage when the disease is suspected:

- in the case of skin symptoms by means of a dermatologist’s report,
- in the case of respiratory complaints by means of an occupational disease notification.

**Clinical picture and differential diagnosis**

In food-processing professions, work related skin symptoms of different kinds are common at the hands, with eczematous skin symptoms predominating. Hand eczema can be irritant, allergic and endogenous in origin. Specific occupational and non-occupational triggers should be clarified by history and epicutaneous testing [41, 245, 255]. 

IgE-mediated contact urticaria to food allergen sources must be distinguished from non-immunologic contact urticaria (e.g., triggered by benzoic acid, sodium benzoate, sorbic acid, abietic acid, nicotinic acid esters, cinnamic acid, cinnamaldehyde, perubalsam) [245]. The latter usually remains confined to the area of contact, whereas systemic forms of manifestation may occur in IgE-mediated contact urticaria [260]. Non-occupational forms of urticaria should be considered for differential diagnosis [261]. 

**Diagnostics**

In cases of suspected IgE-mediated work-related allergic diseases, especially work-related rhinopathy/asthma, a diagnosis should be made early, as long as the patient has not yet left the workplace [248]. 

Stepwise diagnosis includes history, prick test (additionally epicutaneous test in PCD), specific IgE determination and exposure tests [241, 242, 250, 257, 258, 262]. Because extracts for occupationally relevant food allergen sources are often lacking or not sufficiently standardized, in vivo and in vitro diagnostics can be difficult [249, 250]. Diagnostic sensitivity and specificity vary sometimes considerably among currently available occupational allergens depending on the allergen source and test solution [259, 263]. Prick test solutions from different manufacturers should be tested in parallel if available [259]. For the detection of CU and PCD to unstable food allergen sources, a prick-to-prick test with fresh material is recommended [241, 264]. 

Prick tests for the diagnosis of occupational type I allergies should be performed with a metal lancet, if possible using duplicate determinations. If reproducible, wheals with a small wheal diameter (≥ 1.5 mm) should also be considered positive and serologically confirmed if the control is negative [259]. Allergen avoidance and re-exposure under supervision by a physician as well as workplace-based provocation testing may be required to confirm the diagnosis. The specific inhalation provocation test is the gold standard procedure for many occupational asthma triggers [248]. However, a negative result in this test or after workplace exposure is not sufficient to exclude the diagnosis of occupational asthma when the evidence is otherwise good [248, 250, 257]. Further diagnostic measures are provided in the guideline “Prevention of work-related obstructive airway diseases” [248, 257]. 

**Course and therapy**

In occupationally caused IgE-mediated allergies to food components, early allergen avoidance is crucial to prevent increasing symptoms and the development of an OD like BK 5101 (skin symptoms) or an BK 4301 (respiratory symptoms) [248, 256, 265]. Therapeutic measures and the benefit of various management options for work-related allergic rhinopathy and obstructive airway disease are described in the guideline “Prevention of work-related obstructive airway disease” [248]. 

Allergen avoidance through omittment of exposure or the use of appropriate protective equipment can lead to an improvement or healing of IgE-mediated skin manifestations to food allergen sources, but are not always successful [242]. In the food-processing sector, affected individuals with PCD show more severe courses and a less favorable prognosis than patients with skin manifestations at the hands of other etiologies. Significant differences existed with respect to

- the need to consistently wear protective gloves at work,
- the duration of incapacity for work, and
- the frequency of change of profession [246].

If symptom control by allergen avoidance or reduced exposure by technical or organizational measures or personal protective equipment cannot be achieved, there may be an objective necessity to discontinue work in cases of occupationally acquired IgE-mediated food allergy. In addition to the extent of the clinical manifestations [260], the evaluation of the reduction in earning capacity (MdE) also includes the proportion of jobs on the general labor market that are excluded by the allergy [256, 265]. 

It can happen that occupational skin and respiratory symptoms are triggered simultaneously by food allergens. Since this is a uniform allergic disease with symptoms in different organs, this constellation is to be treated as one insured event – based on BK 5101 and BK 4301 – and an overall MdE is to be formed, taking into account the consequences of the allergy [265, 266]. [Table Consensusstatement17]

## Methods report 

Guideline initiation and stakeholder participation. 

The S2k guideline “Management of IgE-mediated food allergy” (AWMF registry number 061-031) was initiated by the German Society of Allergology and Clinical Immunology (DGAKI). The coordination of the guideline project was carried out by Prof. Dr. med. Margitta Worm.****

15 professional societies, professional associations and other organizations participated in the preparation of the guideline and delegated mandate holders for the guideline group (Table 20). Patient interests were represented by the German Allergy and Asthma Association. 

**Formulation of recommendations and structured consensus building**

Draft text and recommendations of the guideline chapters were revised by the authors and subsequently submitted to the guideline group by e-mail. In deriving the recommendations, three levels of recommendation, expressing the strength of the recommendations, were distinguished (Table 1). 

During two online interdisciplinary consensus conferences on December 14, 2020, and January 11, 2021, the recommendations and key messages were consented using a nominal group process. After presentation of the recommendations for consensus, each group member commented on the draft. Dissenting suggestions were noted. This was followed by the steps of row discussion, pre-voting, debate/discussion, and final voting. Each member of the expert group had one vote. A strong consensus (> 95% agreement) was generally sought. If this could not be achieved even after discussion, adoption was by consensus (> 75% agreement). In the case of a recommendation, only “majority agreement” could be achieved (50 – 74% agreement). The corresponding consensus strengths were documented. For those recommendations or key statements for which consensus could not be reached at the consensus conference due to time constraints, a Delphi process was conducted. 

This guideline is intended for all physicians and other health care professionals involved in the acute treatment, diagnosis, and counseling of patients with food allergy. 

**Adoption by the boards of the participating organizations**

On April 22, 2021, the guideline manuscript was sent to the executive boards of all participating professional societies and professional associations as well as the patient organization for their information and request for formal adoption. 

As of June 9, 2021, the approval of the different involved organizations took place. 

**Funding of the guideline**

Funded by the DGAKI. 

**Disclosure and handling of conflicts of interest.**

To disclose potential conflicts of interest, all guideline group members completed the Conflict of Interest Declaration form. The declarations were presented and discussed at the consensus conference. No significant conflicts of interest were identified. 

A summary of the conflict of in terest declaration is available on the AWMF website at http://www.awmf.org/leitlinien/detail/061-031.html. 

**Validity period and update procedure**

Valid until December 31, 2024, the update should be initiated by the responsible persons of the DGAKI, currently guideline coordinator of this guideline Prof. Margitta Worm, MD. 

**Abbreviations Abbreviations:** Abbreviations.

AAAAI	American Academy of Allergy, Asthma and Immunology
AGATE	Working Group on Anaphylaxis – Training and Education
ASA	Acetylsalicylic acid
BAT	Basophil activation test
OD	Occupational disease
CCD	Cross-reactive carbohydrate determinants, cross-reactive carbohydrate side chains
CSACI	Canadian Society of Allergy and Clinical Immunology
DBPCFC	Double-blind placebo-controlled food challenge
DGES	Study on the health of adults in Germany
DELBI	German instrument for methodological guideline evaluation
EAACI	European Academy of Allergy and Clinical Immunology
EGID	Eosinophilic gastrointestinal disorders, eosinophil-associated diseases of the gastrointestinal tract
FDA	Food and drug administration
FPIES	Food protein-induced enterocolitis syndrome, food protein-induced enterocolitis syndrome
GIT	Gastrointestinal tract
HMO	Human milk oligosaccharides
HMW	High molecular weight
IgE	Immunoglobulin E
IgG	Immunoglobulin G
GR	Gastroesophageal reflux
CI	Confidence interval
CU	Contact urticaria
LCPUFA	Long-chain polyunsaturated fatty acids
LY	Life year
LMIV	Food Information Regulation
LoQ	Limit of quantitation
LTP	Lipid transfer protein
NPV	Negative predictive value
NSAID	Non-steroidal anti-inflammatory drug
nsLTP	Non-specific lipid transfer protein
OAS	Oral allergy syndrome
OD	Occupational disease
OIT	Oral immunotherapy
PCD	Protein contact dermatitis
PPI	Proton pump inhibitor
PPV	Positive predictive value
PR-10	Pathogenesis-related protein family 10
RWC	Reduction in earning capacity
SCIT	Subcutaneous immunotherapy
SIT	Specific immunotherapy
SLIT	Sublingual immunotherapy
WDEIA	Wheat-dependent exercise-induced anaphylaxis

**Consensus statements Consensusstatement1:** Consensus statements

The prevalence of food allergy is age-dependent. A study on the prevalence of food allergy in Germany shows a frequency of 4.2% in children and 3.7% in adults.	Strong consensus
IgE-mediated food allergy includes primary (predominantly early childhood) and secondary (predominantly pollen-associated) allergies that vary in severity.	Consensus
Food allergy can severely limit quality of life and in rare cases can be fatal.	Consensus

**Consensus statements Consensusstatement2:** Consensus statements

For prevention of hen’s egg allergy, thoroughly heated (e.g., baked or hard-boiled) but not “raw” hen’s egg (including scrambled egg) should be introduced with complementary feeding and given regularly.	Strong consensus
To prevent peanut allergy, infants with atopic dermatitis in families with regular peanut consumption may consider introducing peanut products in an age-appropriate form (e.g., peanut butter) as part of the complementary food introduction and continue to give them regularly.	Consensus
Before introduction of peanut allergy should be ruled out first, especially in infants with moderate to severe AD.	Consensus

**Consensus statements Consensusstatement3:** Consensus statements

Symptoms of an IgE-mediated food allergy are multifaceted and affect different organ systems (especially skin and oropharyngeal mucosa, gastrointestinal tract, respiratory tract, cardiovascular system).	Strong consensus
For the diagnosis of food allergy, a clear and reproducible association of symptoms with the ingestion of defined foods and improvement of symptoms with avoidance, including in association with IgE sensitization on skin, blood or intestine, etc. In blood or skin IgE-negative patients, local seronegative IgE-mediated allergic reactions are possible, among others.	Strong consensus
In cases of intermittent food tolerability a cofactor should be considered, e.g., cofactor-dependent food allergy such as exercise-induced analphylaxis.	Consensus

**Consensus statement Consensusstatement7:** Consensus statements

Instead of a quantitative IgE result, the severity of a clinical reaction should be determined by history and/or provocation testing. Strong consensus	Strong consensus
Reasonable indications for IgE determination are: - reasonable suspicion of IgE-mediated food allergy, - the specific exclusion of an IgE-mediated food allergy, - a life threatening reaction to food, - suspected sensitization to food,which can not be skin tested - conditions that do not allow skin testing or its evaluation (e.g., urticaria factitia, generalized skin disease, administration of drugs that interfere with skin test results), - very young patients (infants or young children), - an expected diagnostic added value of molecular allergy diagnostics	Strong consensus
Total IgE should be determined as an aid to interpretation.	Consensus
For specific questions, IgE diagnostics with single allergens should be used to detect sensitization	Strong consensus
In vitro diagnostics with single allergens may increase test sensitivity, especially for unstable or underrepresented food allergens	Majority consensus
Sensitization to defined allergen components (see tables in 4.2) may be associated with systemic allergic reactions. Their determination increases analytical specificity compared to food extracts.	Strong consensus

**Table 1.** Strengths of recommendation.

**Recommendation strength**	**Syntax**
Strong recommendation	shall
Recommendation	should
Recommendation open	may

**Consensus statements Consensusstatement4:** Consensus statements

In the case of suspected food allergy, the differential diagnosis should primarily include infections, chronic inflammatory diseases including eosinophilic gastroenteritis and mastocytosis, carbohydrate malabsorption or functional or somatoform disorders.	Strong consensus
For differential diagnosis of suspected food allergy, other diseases should be considered depending on the symptoms and the age of the patient.	Strong consensus
If non-IgE-mediated gastrointestinal intolerance reactions are suspected, a gastroenterologist (or pediatric gastroenterologist) should be involved in the diagnostic work-up.	Consensus

**Table 2.** Comparison of the German recommendations for the prevention of food allergy and possibly other allergic diseases with the EAACI recommendations for the prevention of food allergy in infants and young children.

**Update Guideline Allergy Prevention DGAKI/ GPA 20/21**	**EAACI Recommendation 2020**
**Statement:** During pregnancy and lactation, a balanced, varied diet that meets nutritional needs is recommended. This includes consumption of vegetables, milk/dairy products (including fermented dairy products such as yogurt), fruits, nuts, eggs, and fish. **Recommendation:** Dietary restrictions (avoidance of potent food allergen sources) during pregnancy or lactation should not occur for allergy prevention reasons. (A)	The EAACI Task Force suggests against restricting consumption of potential food allergens during pregnancy or breastfeeding in order to prevent food allergy in infants and young children.
**Statement**: Any breastfeeding has many benefits for mother and child. **Recommendation:** If possible, exclusive breastfeeding should be used for the first 4 – 6 months. (A) Breastfeeding should continue with the introduction of complementary foods. (A)	There is no recommendation for or against using breastfeeding to prevent food allergy in infants and young children, but breastfeeding has many benefits for infants and mothers and should be encouraged wherever possible.
**Recommendation:** Supplemental feeding of cow’s milk-based formula in the first days of life should be avoided if the mother wishes to breastfeed. (B)	The EAACI Task Force suggests avoiding supplementing with cow’s milk formula in breastfed infants in the first week of life to prevent cow’s milk allergy in infants and young children
**Recommendation:** If breastfeeding is not possible or not sufficient, infant formula should be given. For infants at risk, consider whether an infant formula with efficacy demonstrated in allergy prevention studies is available until complementary feeding is introduced. (B)	For infants who need a breastmilk substitute, there is no recommendation for or against the use of regular cow’s milk based infant formula after the first week of life to prevent food allergy. There is no recommendation for or against using partially or extensively hydrolysed formula to prevent food allergy in infants and young children. When exclusive breastfeeding is not possible many substitutes are available for families to choose from, including hydrolysed formulas.
**Recommendation:** Soy-based infant formulas are not suitable for the purpose of allergy prevention and consequently should not be given for this purpose. (A) **Statement:** Soy products can be given separately from the purpose of allergy prevention as part of complementary feeding.**** **Recommendation:** Since there is no evidence of an allergy-preventive effect of other animal milks, such as goat’s milk (not even as the basis of infant formula), sheep’s milk, or mare’s milk, these should also not be given for the purpose of allergy prevention. (B)	The EAACI Task Force suggests against introducing soy protein-based formula in the first six months of life to prevent cow’s milk allergy in infants and young children.
**Statement:** There is evidence that the diversity of the infant’s diet in the first year of life has a protective effect on the development of atopic diseases. A varied diet includes the introduction of fish and a limited amount (up to 200 ml per day) of milk or natural yogurt and hen’s egg as part of complementary feeding.**** **Recommendation:** Depending on the readiness of the infant, complementary feeding should begin no earlier than the beginning of the fifth month of life and no later than the beginning of the seventh month of life. (B) There is no evidence for a preventive effect of dietary restriction by avoiding potent food allergen sources in the first year of life. Therefore, it should not be done. (A)	
**Recommendation:** For prevention of egg allergy, heated (e.g., baked or hard-boiled) but not „raw“ eggs (including scrambled eggs) should be introduced with complementary feeding and given regularly. (B)	The EAACI Task Force suggests introducing well-cooked hen’s egg, but not pasteurised or raw egg, into the infant diet as part of complementary feeding to prevent egg allergy in infants.
**Recommendation:** To prevent peanut allergy, consider introducing peanut products in an age-appropriate form (e.g., peanut butter) as part of complementary feeding in infants with atopic dermatitis in families with regular peanut consumption. (C)**** **Recommendation:** Peanut allergy should be ruled out first, especially in infants with moderate to severe AD. (A**)**	In populations where there is a high prevalence of peanut allergy, the EAACI Task Force suggests introducing peanuts into the infant diet in an age-appropriate form as part of complementary feeding in order to prevent peanut allergy in infants and young children.
**Background:** Due to the heterogeneity of studies, no conclusive recommendation can be made on the supplementation of Ω-3 LCPUFAs for pregnant women, breastfeeding women, and infants for allergy prevention.**** **Statement:** Some studies show that a low supply of Ω-3 LCPUFAs in pregnant women, breastfeeding women and infants is associated with a higher risk of allergic diseases in the child, especially asthma and wheezing, and that this risk can be reduced by supplementation of Ω-3 LCPUFAs (1++ to 2++).	There is no recommendation for or against vitamin supplementation or fish oil supplementation in healthy pregnant and/or breastfeeding women and/or infants to prevent food allergy in infants and young children.
**Statement:** Data from partly large, randomized, double-blind intervention studies consistently show no preventive effects of pre- and probiotics for the **endpoints allergic rhinitis (AR) and bronchial asthma**. The vast majority of current intervention studies also show no preventive effect for atopic eczema after administration of prebiotics and/or probiotics.**** **Recommendation:** Prebiotics and/or probiotics should not be given to pregnant women or infants, even as part of infant formula, for allergy prevention purposes. (A)	There is no recommendation for or against prebiotics, probiotics or synbiotics for pregnant and/or breastfeeding women and/or infants alone or in combination with other approaches to prevent food allergy in infants and young children.
**Background:** From the point of view of the guideline group, despite heterogeneous interventions in the different studies, it has not been shown that primary prevention in infants with atopic family history can be achieved by daily refatting whole body treatment of healthy skin. **Statement:** At the present time, based on the available evidence, no recommendation can be made for daily re-lubrication of healthy infant skin with the aim of primary prevention of eczema and allergies – even in families with an increased risk of allergies. **Recommendation:** Infants and children with visibly dry skin should be creamed regularly – also with the aim of preventing eczema and allergies. (Expert opinion)	There is no recommendation for or against using emollients as skin barriers to prevent food allergy in infants and young children.

**Table 3.** Symptoms of food allergy.

**Target organ**	**Symptoms**
Systemic; Circulation	Anaphylaxis
	Hypotension, shock
	Tachycardia (rarely bradycardia in anaphylaxis)
	Drowsiness, dizziness
	Syncope
Skin (transient)	Erythema („flush“)
	Eczema (worsening)
	Urticaria
	Itching
	Angioedema
	Exanthema
Eye	Itching
	Redness (conjunctival injections)
	lacrimation
	periorbital edema
Upper respiratory tract	Nasal congestion
	Itching
	Rhinorrhea
	Laryngeal edema, stridor
	Hoarseness
	Dry cough
Lower respiratory tract	Cough
	Thoracic tightness
	Heaviness, shortness of breath (dyspnea)
	Whistling breath sounds (wheezing)
	Cyanosis
Oropharyngeal	Swelling of lips, tongue and/or palate (angioedema)
	Oral and/or pharyngeal itching (pruritus)
	Tongue swelling
Gastrointestinal tract	Nausea
	Vomiting
	Colicky abdominal pain
	Gastroesophageal reflux
	Diarrhea

**Table 4.** Symptoms of delayed reaction or in case of chronic exposure.

Nausea
Vomiting
Abdominal pain
Gastroesophageal reflux, dysphagia, and bolus events
Inappetence and food refusal
Diarrhea, malassimilation, enterocolitis
Hematochezia (blood in stool)
Failure to thrive and weight loss

**Table 5.** Manifestations and differential diagnoses of food allergy. Modified according to [24].

**Immunopathology**	**Disease**	**Clinical characteristics**	**Typical age group**	**Prognosis**
IgE- mediated	Acute urticaria/ angioedema	Triggered by ingestion or direct skin contact	Children > adults	Depending on the triggering food
	Rhinoconjunctivitis/asthma bronchiale	Accompanied by food protein allergic reactions, rarely isolated respiratory symptoms (exception: inhalation exposure to aerosol of food protein, often occupational)	Infant > adult, except occupational	Dependent on the triggering food substance
	Anaphylaxis	Rapidly progressive multisystem reaction	Any age	Depending on triggering food and underlying disease
	Delayed food-induced anaphylaxis to mammalian meat [267]	Anaphylaxis three to six hours after ingestion; triggered by antibodies to galactose-α-1,3-galactose	Adults > children	Unclear
	Food-dependent, risk factor-dependent anaphylaxis	Food triggers anaphylaxis only if augmentation factors such as exertion, but also alcohol or acetylsalicylic acid (ASA) are present before or after food ingestion	Onset in late childhood/adulthood	Probably permanent
	Secondary cross-allergy (mainly pollen-associated food allergies)	Oropharyngeal itching; mild edema confined to oral cavity, less frequently urticaria perioral or generalized, Respiratory symptoms (cough); – rarely systemic reactions (incl. anaphylaxis) in some pollen-associated allergies	Onset after manifestation of pollen allergy (adult > young child)	May persist; may vary with seasons
	Gastrointestinal allergic immediate reaction (allergic esophagitis, gastritis, enteritis or colitis)	After ingestion, – depending on resorption and/or reaction site – occurring bolus sensation, vomiting, nausea, or abdominal colic, diarrhea or enterocolitis	Any age	Depending on the triggering food
Mixed IgE- and cell- mediated	Atopic eczema/dermatitis	Associated with food in 30 to 50% [268] of children with moderate/severe eczema	Infant > child > adult	Usually development of tolerance
	Eosinophil-associated gastrointestinal inflammatory disease (EGID)	Symptoms vary; likely persistent depending on part of gastrointestinal tract affected and degree of eosinophil inflammation	Any age	Unclear
Cell- mediated	Food protein-induced proctitis/proctocolitis	Mucopurulent, bloody stools in infants	Infants	Usually tolerance development
	Food protein-induced enterocolitis syndrome (FPIES)	Acute exposure: severe manifestation with vomiting, (bloody) diarrhea and exsiccosis to shock; chronic exposure: vomiting, diarrhea, failure to thrive, lethargy, Re-exposure after abstinence: vomiting, diarrhea, hypotension one to three hours after ingestion	Infants – young children, less frequently adults [269]	Usually development of tolerance
	Food protein-induced enteropathy	Diarrhea, vomiting, failure to thrive, edema; no colitis	Infants – young children > adults	Usually development of tolerance in children
	Celiac disease	Multiple manifestations, mono-, oligo- and polysymptomatic, triggered by gluten in case of genetic predisposition	Persistent at any age (lifelong strict gluten avoidance required)	Permanent
Non-allergic (non-immunological intolerance)	Carbohydrate mal-assimilation/absorption (lactose, fructose, sorbitol, rarely: sucrose, glucose-galactose)	Diarrhea (osmotic), meteorism, abdominal pain one to four hours after ingestion, constipation also possible	Lactase deficiency typically from school age, otherwise any age fructose mal-absorption/sorbitol: any age, very rare: congenital lactase deficiency, glucose-galactose intolerance, sucrose-isomaltase malabsorption	Mostly persistent (lactose, glucose-galactose); fructose, sorbitol

**Consensus statements Consensusstatement6:** Consensus statements

A detailed medical history should be the basis for the diagnosis of food allergy.	Strong consensus
The structured history should consider triggers, time course, symptoms, severity, reproducibility, risk and augmentation factors, family history, concomitant diseases and other allergic diseases.	Strong consensus
For chronic symptoms, a diet and symptom diary is useful.	Strong consensus

**Consensus statements Consensusstatement5:** Consensus statements

Specific testing for IgE sensitization should be guided by the medical history.	Strong consensus
Detection of IgE sensitization to foods and aeroallergens should be by specific IgE determination and/or skin prick testing.	Consensus
Specific IgE determination and skin prick testing support the diagnosis of food allergy in the context of history and/or food provocation.	Strong consensus
Detection of sensitization by specific IgE determination or skin prick test does not prove the clinical relevance of the food tested and should not alone lead to therapeutic elimination.	Strong consensus
Lack of evidence of sensitization (negative specific IgE/skin prick test) often, but not certainly, excludes clinically relevant IgE-mediated food allergy.	Consensus

**Table 6.** Procedure for taking medical history.

**Medical history**	
Self history	Known allergic diseases Medication Physical exertion Acute infectious diseases Psychological stress
Family history	Allergic diseases in first-degree relatives
Symptoms or specific triggers	When Where By what How long How often Repeatedly
Nutritional history	Record dietary restrictions and extent, tolerance of foods with proven sensitization
Dietary and symptom diary	Documentation of diet and symptoms

**Table 7.** Important allergen sources in food allergies in children and adults.

**Children**	**Adolescents and adults**
Cow’s milk	Pollen-associated food allergen sources (e.g., stone and pome fruits, nuts, soy, celery, carrot)
Hen’s egg	Nuts and oilseeds (e.g., sesame)
Peanut	Peanut
Wheat	Fish and shellfish
Nuts	Cow’s milk*, Hen’s egg*
Soy*	Latex-associated food allergen sources* (e.g., banana, avocado, kiwi, fig)
Fish*	Mammalian meat*

*Rare.

**Table 8.** List of definitions and abbreviations.

Allergen	Molecule (protein, e.g., major allergen Gad c 1 from cod, rarely carbohydrate component) that can trigger an allergic immune response.
Allergen extract	Mixture of allergenic and non-allergenic components extracted from an allergen source (e.g., fish allergen extract)
Allergen source/carrier	Origin/starting material of allergens (e.g., fish).
α-Gal	Galactose-α-3-galactose, a disaccharide as a cause of anaphylaxis to mammalian meat, gelatin, and biologics
Ara h 2	2S albumin, a storage protein of peanut, associated with systemic reactions in peanut allergy
Api g 1	Celery allergen with homology to Bet v 1, responsible for birch pollen-associated cross-reactions
Bet v 1	Immunodominant major allergen in birch pollen (*Betula verrucosa*)
Bet v 2	Birch pollen profilin, minor allergen, which as a panallergen in numerous pollens and plant foods (e.g., melon) can be responsible for cross-reactions and thus complicates diagnosis
CCD	Cross-reactive carbohydrate determinants. They represent epitopes of N-glycans, and less often of O-glycans, as panallergens, are responsible for a pronounced cross-reactivity.
Cor a 1.04	Hazelnut allergen with homology to Bet v 1, responsible for birch pollen-associated cross-reactions
Dau c 1	Carrot allergen with homology to Bet v 1, responsible for birch pollen-associated cross-reactions
Gad c 1	Major cod allergen (Ca2+ transport protein, parvalbumin, major fish allergen)
Gly m 4	Soy allergen with homology to Bet v 1, responsible for birch pollen-associated, sometimes severe cross-reactions
Cross-reactive	Similarity-induced immunological reaction with molecular structures that were not responsible for the original sensitization
LTP	Lipid transfer proteins; thermo- and digestion-stable allergens of plant origin
Mal d 1	Apple allergen with homology to Bet v 1, responsible for frequent birch pollen-associated, mostly oropharyngeal cross-reactions
MUXF3	Designation of the structure of a carbohydrate side chain of plant glycoproteins and allergens that can potentially be bound by IgE antibodies, corresponds to a specific type of CCD (see above)
Oleosins	Lipophilic and thermostable allergens in nuts and oilseeds.
Pen a 1	Tropomyosin (muscle structure protein) of shrimp with homologous proteins in other arthropods and cause of cross-reactions
PR-10	„Pathogenesis-related protein family 10“; Bet-v-1 homologous proteins with defense function in plants (including tree pollen, food)
Pru p 3	Lipid transfer protein in peach responsible for systemic reactions in patients in the Mediterranean region
Recombinant	Produced with the aid of genetically modified (micro)organisms
Recombinant allergen	Allergenic protein often produced in *Escherichia coli *without the carbohydrate side chains found in native allergens
Sensitization	Allergenicity (only relevant in case of corresponding symptoms)
Tri a 19	ῳ-5-gliadin in wheat, responsible for systemic reactions and effort-dependent anaphylaxis in wheat allergy

**Table 9.** Selected food allergens and their sources of plant origin^a,b^.

	**Protein families **					**Storage proteins (protein families, structure)**		
						**Prolamins**	**Cupins**	
	**Bet-v-1 homologue**	**LTP**	**Profilins**	**Thaumatins**	**Oleosins**	**2S Albumins**	**7/8S-Globulins (Vicilin)**	**11S-Globulins (Legumin)**
Apple	**Mal d 1**	**Mal d 3**	Mal d 4	Mal d 2				
Peanut	**Ara h 8**	**Ara h 9 ** **** Ara h 16 Ara h 17	Ara h 5		Ara h 10 Ara h 11 Ara h 14 Ara h 15	**Ara h 2 ** **Ara h 6 ** Ara h 7	**Ara h 1**	**Ara h 3**
Hazelnut	**Cor a 1**	**Cor a 8**	Cor a 2		Cor a 12 Cor a 13	**Cor a 14**	Cor a 11	**Cor a 9**
Carrot	Dau c 1	Dau c 3	Dau c 4					
Cherry	Pru av 1	Pru av 3	Pru av 4	Pru av 2				
Peach	**Pru p 1**	**Pru p 3**	**Pru p 4**					
Celery	**Api g 1**		Api g 4					
Sesame					Ses i 4 Ses i 5	**Ses i 1** Ses i 2	Ses i 3	Ses i 6 Ses i 7
Soybean	**Gly m 4**	Gly m 1	Gly m 3			Gly m 8	**Gly m 5**	**Gly m 6**
Wheat		**Tri a 14**	Tri a 12			**Tri a 19** (ω-5-Gliadin)		

^a^Allergen sources (left column) with individual allergens (table columns) and their protein families (header). ^b^Bold print: already available for in vitro diagnostics, normal print: not yet available for differentiating diagnostics

**Table 10.** Selected food allergens of animal origin^a,c^.

	**Protein families**			
	**Parval- bumins**	**Tropo- myosins**	**Lysozyms/ α-Lactal- bumins**	**Other proteins (various families)**
Hen’s egg			**Gal d 4** (Lysozym C)	**Gal d 1** (ovomucoid, trypsin inhibitor) **Gal d 2** (ovalbumin, Serpin) **Gal d 3** (ovotransferrin, Conalbumin)
Fish	**Gad c 1 ** **Cyp c 1**	Ani s 3^b^		
Crustaceans/mollusks	Hom a 6	Cha f 1 Hom a 1 Met e 1 **Pen a 1**		
Cow’s milk			**Bos d 4** (α-lactal- bumin)	**Bos d 5** (β-lactoglobulin, lipocalin) **Bos d 6** (bovine thyroglobulin) **Bos d 8** (Casein)
Mammalian meat				**Galactose-α-1,3-Galactose (α-GAL) (diasaccharide on proteins (**bovine thyro- globulin****and **glycolipids)**

^a^Allergen sources (left column) with individual allergens (table columns) and their protein families (header row). ^b^Due to infestation with the herring worm (*Anisakis*), severe allergic reactions have been described after consumption of infested fish. ^c^ **Bold**: already available for in vitro diagnostics, normal/non-bold: not yet available.

**Table 11.** Open Access internet resources/databases and information on molecular allergology [270].

**Web-Link**	**Brief description**
www.allergen.org	Official database of the WHO/IUIS Allergen Nomenclature Sub-committee with simplified search function
www.allergenonline.org	Food Allergen Database of the University of Nebraska at Lincoln, Food Allergy Research and Resource Program (FARRP); carefully maintained entries organized by taxonomic affiliation of allergen sources
www.allergome.org	Largest database on allergen molecules, initiated by the Italian allergist Adriano Mari and his team; some entries of identified single allergens before their official naming
www.meduniwien.ac.at/allergens/allfam/	Database on allergen families (protein families) of the Medical University of Vienna, Institute of Pathophysiology and Allergy Research in the Center for Pathophysiology, Infectiology and Immunology
www.thermofisher.com/diagnostic-education/hcp/de/de/resource- center/allergen-encyclopedia.html	Extensive database for allergen extracts and molecules with additional clinical information from a diagnostic manufacturer

**Table 12.** Examples of clinical patterns and molecular diagnostic recommendations [59].

**Clinical picture**	**Clinical suspicion**	**IgE diagnostics**
Anaphylaxis after exertion	Exertion-dependent wheat allergy	Tri a 19 (ω-5-gliadin)
„Cat-pork syndrome“	Allergy to animal serum albumin	Fel d 2 or Bos d 6
Delayed meat allergy (e.g., urticaria)	Sensitization to galactose-α-1,3-galactose (α-GAL)	α-GAL (bovine thyroglobulin)
Allergy e.g., to grapes, berries, lettuce	Sensitization to lipid transfer protein (LTP)	Pru p 3 (peach LTP)
Oral allergy syndrome (OAS) frequently to nuts, pome and stone fruits, etc., possibly systemic reactions to soy (native)	Sensitization to Bet v 1 homologue (PR 10 proteins)	Bet v 1 and possibly Gly m 4
OAS after atypical plant foods (melon, exotics such as lychee, citrus fruits)	Sensitization to profilins	Pru p 4 (or Bet v 2, Phl p 12, Hev b 8)

**Table 13.** Barriers for the evaluation of specific IgE results.

**Technical and methodological errors** (reasons for false-positive and false-negative results)
Poor quality of reagents (e.g., allergen extracts or their extraction, coupling and stability) laboratory errors
**Interpretation errors** (reasons for clinically irrelevant results)
Strongly increased total IgE with multiple sensitizations high sensitivity of detection cross-reactive IgE antibodies

IgE = Immunoglobulin E.

**Consensus statements Consensusstatement11:** Consensus statements

Because of the natural history of cow’s milk, hen’s egg, wheat, and soy allergy in children, oral food provocations should be repeated at regular intervals (e.g., every 6, 12, or 24 months) to assess tolerance development.	Strong consensus
In children with peanut and primary tree nut allergy and allergy to fish and oilseeds, follow up provocation testing should be performed within longer intervals (e.g., every 3 – 5 years).	Consensus
The course of sIgE antibodies over time is a useful parameter to assess whether repetitive provocation is required.	Expert opinion

**Consensus statements Consensusstatement12:** Consensus statements

**Acute therapy**	
Patients at risk for anaphylaxis should receive emergency medication for self-administration, including an epinephrine autoinjector.	Strong consensus
Severe allergic reactions to food should be treated primarily with intramuscular epinephrine.	Strong consensus
Antihistamines should be used for acute cutaneous symptoms, especially urticarial reactions and mucosal reactions.	Strong consensus
Prophylactic use of antihistamines to prevent food allergic reactions is not recommended.	Consensus
**Continuous therapy**	
Cromoglycic acid and ketotifen did not show a consistent therapeutic effect when unselected patient cohorts were treated, therefore currently no consistent treatment recommendation is possible for patients. If gastrointestinal symptoms are present, individual treatment decisions and monitoring are recommended.	Consensus

**Consensus statements Consensusstatement9:** Consensus statements

Oral food provocation (especially double-blind placebo-controlled) is the gold standard in the diagnosis of IgE-mediated food allergy.	Strong consensus
If there is evidence of augmentation factors, these should be taken into account during provocation.	Strong consensus
Food provocations should be performed when indicated to confirm or exclude allergy. Provocation provides the basis for a safe food ingestion, allows counseling regarding appropriate allergen avoidance, and provides an assessment of the risk for severe reactions (anaphylaxis).	Consensus
A negative oral food provocation should be followed by repetitive cumulative administration of the tested food in an age- and daily-adapted amount no earlier than the next day to confirm clinical tolerance.	Strong consensus
Oral food provocations should be performed in specialized facilities where emergency measures are immediately available. In addition, for provocation with a high risk of severe allergic reactions, intensive medical support should be available.	Strong consensus

**Consensus statements Consensusstatement8:** Consensus statements

The preferred skin test method for the diagnosis of IgE-mediated food allergy is the skin prick test.	Strong consensus
Scratch testing, friction testing, intradermal testing, and closed epicutaneous testing (atopy patch testing) are not recommended for routine diagnosis of food allergy.	Consensus
Depending on the stability and safety of the food allergens, testing should be performed with commercial test solutions or native foods.	Strong consensus

**Consensus statement Consensusstatement10:** Consensus statement

Diagnostic testing procedures such as bioresonance, electroacupuncture, kinesiology, cytotoxic food tests as well as IgG/IgG4 determinations and lymphocyte transformation tests with food should not be performed for the diagnosis of food allergy or intolerance	Strong consensus

**Rationale of food provocation Rationaleoffoodprovocation:** Rationale of food provocation

**Indication**	**Rationale**
Common indications for oral food provocation	1. Basis for informed treatment planning, including a food- and nutrient-based recommendation
	2. Basis for informed treatment planning, including a food- and nutrient-based recommendation
	3. Suspected allergic reaction where the trigger remains unclear despite allergy diagnosis (reaction after compounded meal)
	4. Evidence of sensitization, but the corresponding food has never been consumed or has only been consumed in small amounts
	5. Confirmation of clinical relevance after improvement of clinical symptoms, e.g., atopic dermatitis, under elimination diet
	6. Proof of a natural development of tolerance
	7. Evidence of efficacy of a causal therapy, e.g., oral immunotherapy in the context of clinical research
Strong consensus	

**Table 14.** Overview of the suitability of prick test materials [271]^c^.

	**Commercial extract**	**Suitable for native test** ^a^	**Limited suitability for native test** ^b^
**Food of animal origin**			
Fish	+	+	
Meat	(+)	+	
Chicken egg	+	+	
Seafood and snails	+	+	
Milk	+	+	
**Food of vegetable origin**			
Pineapple			+
Apple		+	
Cereals	(+)	+	
Strawberries			+
Peanuts	+	+	
Spices			+
Hazelnuts	+	+	
Carrot		+	
Kiwi			+
Lychee		+	
Mango		+	
Oilseeds (e.g., poppy, sesame)		+	
Peach		+	
Celery	(+)	+	
Mustard			+
Soy	(+)	+	
Tomato			+
Grape		+	
Sugar snap pea		+	

^a^Ideally control subjects should be tested because of possible irritant components (testing of control subjects with not approved test preparations is not legal in Germany according to AMG). ^b^Higher irritant potential. ^c^Data on the quality of commercial extracts are only available for individual food prick test solutions from individual manufacturers [271], therefore this table can only provide limited information. It does not allow extrapolation to the performance of test allergens of the same allergen sources from other manufacturers.

**Table 15.** Procedure for provocation tests.

**Design of the provocation open vs. blinded (single or double blind) titrated vs. one-step**		**The design should be selected according to indication and purpose of provocation**
Preparation of the provocation meal		The provocation meal should include, as realistically as possible, the usual edible form of the food that triggered the reaction. A common target amount is 3 – 5 g of the food protein. Processing of the food and incorporation into a matrix can significantly affect allergenicity, e.g., raw or baked egg. In provocations to confirm pollen-associated food allergy, fresh fruits and vegetables should be used if possible, as the triggering proteins are usually heat-labile.
Choice of matrix		Clear care should be taken to ensure that no other allergens to which the patient reacts are included in the meal. As few ingredients as possible should be used. For placebo meals, the sensory characteristics should be as close as possible to those of the test food.
Dosage	Number of doses	In most cases, a titration in seven semi-logarithmic steps should be chosen. If negative provocation is expected and there are no safety concerns, a single dose may be appropriate.
	Initial Dose	In clinical practice, an initial dose of 3 mg of food protein is appropriate for most foods. Smaller doses should be selected for threshold dose provocations and high-risk patients.
	Maximum dose	According to an age-adjusted portion, 3 g of food protein is suitable for most foods.
	Cumulative total dose	A cumulative total dose should be administered the next day or another day, as some patients do not respond until repetitive administration.
	Time interval between doses	20 – 30 minutes, but should be adjusted according to history.

**Table 17.** Lifetime prevalence of food allergy by self-assessment or food provocation. Importance of spontaneous remission in infancy.

	**Lifetime prevalence (self-assessment; 95% CI) [272]**	**Lifetime prevalence (food provocation; 95% CI) [272]**	**Spontaneous remission (by LY)**	**Reference**
**Cow’s milk**	6.0% (5.7 – 6.4)	0.6% (0.5 – 0.8)	50% (5^th^ LY) 57% (2^nd^ LY) 57% (5^th^ LY)	[112] [113] [273]
**Chicken egg**	2.5% (2.3 – 2.7)	0.2% (0.2 – 0.3) 2.01% (1.04 – 3.5)	50% (6^th^ LY) 47% (2^nd^ LY) 49% (2^nd^ LY)	[24, 115, 116] [117, 274]
**Wheat**	3.6% (3.0 – 4.2)	0.1% (0.01 – 0.2)	29% (4^th^ LY), 56% (8^th^ LY), 65% (12^th^ LY) 70% (14^th^ LY)	[118]
**Soy**	−	0.3% (0.1 – 0.4)	25% (4^th^ LY), 45% (6^th^ LY), 69% (10^th^ LY)	[275]
**Peanut**	0.4% (0.3 – 0.6)	0.2% (0.2 – 0.3)	0 – 57% 22% (4^th^ LY)	[120, 121, 122] [125]
**Fish**	2.2% (1.8 – 2.5)	0.1% (0.02 – 0.2%)	0 – 20% (10^th^ LY)	[127, 276]
**Crustaceans**	1.3% (0.9 – 1.7)	0.1% (0.06 – 0.3)	0%	[127]

**Table 16.** Typical first onset of food allergy.

**First year of life**	**Toddler preschool age**	**Adolescent and adult age**
- Cow’s milk - Hen egg - Wheat - Soy - Peanut - Nuts - Fish	- Peanut, other legumes (e.g., lentils/chickpeas) - Nuts - Fish	- Pollen-associated food allergen sources - Cofactor-dependent food allergy (e.g., wheat) - Crustaceans - Fish

**Consensus statements Consensusstatement13:** Consensus statements

An appropriate elimination diet is recommended as a mainstay of food allergy management.	Strong consensus
An elimination diet should be based on sound allergy diagnostics. The scope and indication should be re-evaluated regularly.	Strong consensus
Individuals with food allergy who are on a long-term elimination diet should be counseled by an allergy-experienced dietitian.	Strong consensus
Patients should be educated about allergen labeling (according to the Food Information Regulation) and existing gaps.	Strong consensus
Extensive protein hydrolysates or alternatively amino acid formulas are recommended in cases of existing cow’s milk allergy, especially in infants and possibly toddlers.	Strong consensus
In the presence of an existing cow’s milk allergy, soy-based infant formulas are second-choice cow’s milk substitutes and are not recommended for infants younger than 12 months of age. Soy-containing foods used as milk substitutes beyond this are not affected by this restriction.	Strong consensus

**Consensus statements Consensusstatement16:** Consensus statements

Hen’s egg allergic children shall be vaccinated as other children according to STIKO. Deferral is not necessary.	Strong consensus
Hen’s egg-allergic children shall be vaccinated against MMR varicella like other children according to STIKO; vaccination in a clinic or special outpatient clinic/practice is not necessary.	Strong consensus
Influenza vaccination shall be given to hen’s egg allergic outpatients in practices, provided there is sufficient experience in emergency management of allergic/anaphylactic reactions. In patients who have experienced severe anaphylaxis, influenza vaccination may be monitored as an inpatient if necessary.	Strong consensus
A yellow fever vaccination contains relevant amounts of hen’s egg protein and should only be performed in hen´s egg allergic patients after careful benefit-risk assessment. If necessary the vaccination should be performed in a day hospital, specialized practice or clinic with appropriate expertise regarding anaphylaxis management.	Strong consensus

**Consensus statements Consensusstatement17:** Consensus statements

Diagnostic work-up of suspected IgE-mediated work-related allergic diseases should be performed early, as long as the patient has not yet left the workplace, in order to be able to perform, among other measures, workplace-related investigations and exposure tests as needed, in addition to specific step-by-step diagnostics.	Strong consensus
Even in cases of occupational food allergy, allergen avoidance should be the primary goal, possibly in the form of exposure avoidance through appropriate protective measures. If this is not possible, the necessity of cessation of the activity should be considered.	Strong consensus

**Table 18.** Forms, clinic, and characteristics of occupational food allergies.

**Immunopathology**	**Disease/symptoms**	**Clinical characteristics**	**Typical age group**	**Prognosis**
IgE-mediated	Contact urticaria syndrome (stages I – IV)	Predominantly occupationally triggered by cutaneous contact	Adults, occupationally exposed	Depending on the triggering food and possible avoidance measures
	Occupational obstructive respiratory diseases caused by allergens (incl. allergic rhinopathy)	Predominantly occupational respiratory symptoms caused by inhalation exposure to allergens	Adults, occupationally exposed persons	Depending on the triggering food and possible avoidance measures
Mixed IgE- and cell-mediated	Protein contact dermatitis	Predominantly occupational lesions on the hands triggered by cutaneous contact	Adults, occupationally exposed	More severe effects and less favorable prognosis than occupational skin eruptions of other etiologies
Non-immunological	Non-immunological contact urticaria	Predominantly occupationally triggered lesions on the hands induced by cutaneous contact with benzoic acid, sodium benzoate, sorbic acid, abietic acid, nicotinic acid esters, cinnamic acid, cinnamaldehyde and perubalsam	Adults, occupationally exposed	In contrast to IgE-mediated contact urticaria usually limited to contact area

**Table 19.** Allergen profiles and occurrence as occupational allergens (examples).

**Allergen source**	**Relevant allergens when consumed**	**Occupational allergens**	**Occupation**	**Reference**
Wheat	ω-5-Gliadin (Tri a 19), and other allergens: „wheat-dependent, exercise-induced anaphylaxis“ (WDEIA); Profilin (Tri a 12), nsLTP (Tri a 14); agglutinin isolectin 1 (Tri a 18), ω-5-gliadin (Tri a 19), γ-gliadin (Tri a 20), thioredoxin (Tri a 25), „high-molecular-weight“(HMW)-glutenin (Tri a 26) and other	α-Amylase trypsin inhibitors (e.g., Tri a 28, Tri a 29.0101, Tri a 29.0201, Tri a 30, Tri a 15); „thiol reductase“ (Tri a 27); thioredoxin (Tri a 25), triose phosphate isomerase, α-/β-Gliadin, 1-cysperoxiredoxin (Tri a 32), dehydrin (Tri a DH, serpin, glycerinaldehyde-3-phosphate dehydrogenase (GA3PD), ω-5-gliadin (Tri a 19), nsLTP (Tri a 14), Acyl-CoA-oxidase, fructose-bisphosphat aldolase, serine protease inhibitor-like protein (Tri a 39), and other	Bakers	[249, 250, 252, 262]
Cattle	Beef (meet): Bos d 6 and α-GAL	Dander: Bos d 2 (Lipocalin)	Farmers, cooks	[277] [243]
Soy	Gly m 4 (PR-10-homologue), Gly m 5 (β-conglycinin), Gly m 6 (glycinin) et al.	Soy flour: high molecular weight allergens (Gly m 5 and 6)	Bakers	[278, 279]
		Dust containing soy: low molecular weight allergens (Gly m 1 and Gly m 2)	Dockworkers	
Fish	Parvalbumin e.g. Gad m 1.0101 Gad m 1.0102 Gad m 1.0201 Gad m 1.0202 Sal s 1.0101 Enolase e.g. Gad m 2.0101 Sal s 2.0101 Aldolase Gad m 3.0101 Sal s 3.0101	Skin and inhalation Parvalbumin, glyceraldehyde-3- phosphate dehydrogenase	Fish processing industry, cooks	[280, 281, 282]

nsLTP = non-specific lipid transfer protein.

**Table 20.** Organizations involved.

**Organization**	**Representative**
Medical Association of German Allergists (AeDA)	Dr. med. Katja Nemat
Working Group Dietetics in Allergology (ak-dida)	Dr. rer. medic. Imke Reese
Professional Association of Pediatricians and Adolescents (BVKJ)	Dr. med. Peter J. Fischer
German Allergy and Asthma Association (DAAB)	Sabine Schnadt
German Dermatological Society (DDG)	Prof. Dr. med. Regina Treudler Prof. Dr. med. Knut Brockow
German Society for Allergology and Clinical Immunology (DGAKI)	Prof. Dr. med. Margitta Worm Prof. Dr. med. Uta Jappe Prof. Barbara Ballmer-Weber Prof. Dr. med. Thomas Werfel Prof. Dr. med. Torsten Zuberbier Prof. Dr. med. Joachim Saloga Prof. Dr. med Jörg Kleine-Tebbe Prof. Dr. med. Eckard Hamelmann
German Society for Gastroenterology, Digestive and Metabolic Diseases (DGVS)	Prof. Dr. med. Stephan C. Bischoff Prof. Dr. med. Martin Raithel
German Society of Otorhinolaryngology, Head and Neck Surgery (DGHNO)	Prof. Dr. med. Ludger Klimek Prof. Dr. med. Martin Wagenmann
German Society for Pediatric and Adolescent Medicine (DGKJ)	Prof. Dr. med. Berthold Koletzko
Society for Pediatric Allergology and Environmental Medicine (GPA)	Prof. Dr. med. Kirsten Beyer Dr. med. Lars Lange
German Society for Pneumology and Respiratory Medicine (DGP)	Dr. med. Ute Lepp Prof. Dr. med. Jens Schreiber
German Contact Dermatitis Society (DKG) in the German Dermatological Society (DDG)	Prof. Dr. med. Vera Mahler
Austrian Society of Allergology and Immunology (ÖGAI)	Prof. Dr. med. Zsolt Szépfalusi Prof. Dipl. Ing. Dr. Barbara Bohle
Professional Association of Oecotrophology e.V. (VDOE)	Dipl. oec. troph. Christiane Schäfer
Society for Pediatric Pneumology (GPP)	Prof. Dr. med. Susanne Lau
Society of Pediatric Gastroenterology and Nutrition (GPGE)	Dr. med. Martin Claßen

**Consensus statements Consensusstatement14:** Consensus statements

**Primary food allergy**	
Children between 4 and 17 years of age with a confirmed diagnosis of systemic peanut allergy should be offered oral immunotherapy with an approved preparation, taking into account an individual benefit-risk assessment.	Strong consensus
**Pollen-associated food allergy**	
Pollen-associated food allergy may improve with subcutaneous or sublingual immunotherapy with pollen allergens. Such treatment may be considered only if there is a concurrent indication for treatment of pollen-related respiratory symptoms.	Strong consensus
In pollen-associated food allergy, oral immunotherapy with food allergen sources should only be used in the context of controlled trials.	Strong consensus

**Consensus statements Consensusstatement15:** Consensus statements

Patients, their relatives and caregivers should be informed about foods to avoid and receive practical advice on avoidance measures, recognition and self-management of allergic reactions.	Strong consensus
Patients or those responsible for their medical care (e.g., parents) should receive practical instruction (with AAI trainer) in the use of the emergency kit, including the epinephrine autoinjector. If possible, patients or their relatives, caregivers, and other relevant persons should be offered training in the use of the emergency kit including epinephrine autoinjector.	Strong consensus
Patients should be advised to contact an appropriate patient organization.	Consensus
Food allergic patients at risk of anaphylaxis should receive an anaphylaxis ID card and or they or their caregivers should attend patient/ or parent education.	Strong consensus
